# Phylogeny and Expression Analyses Reveal Important Roles for Plant PKS III Family during the Conquest of Land by Plants and Angiosperm Diversification

**DOI:** 10.3389/fpls.2016.01312

**Published:** 2016-08-30

**Authors:** Lulu Xie, Pingli Liu, Zhixin Zhu, Shifan Zhang, Shujiang Zhang, Fei Li, Hui Zhang, Guoliang Li, Yunxiao Wei, Rifei Sun

**Affiliations:** ^1^Department of Chinese Cabbage, Institute of Vegetables and Flowers, Chinese Academy of Agricultural SciencesBeijing, China; ^2^College of Biological Sciences and Biotechnology, Beijing Forestry UniversityBeijing, China; ^3^College of Horticulture and Landscape Architecture, Hainan UniversityHaikou, China

**Keywords:** *PKS III* multigene family, CHS, STS, phylogenetic reconstruction, functional diversification, gene expression, *cis*-elements

## Abstract

Polyketide synthases (PKSs) utilize the products of primary metabolism to synthesize a wide array of secondary metabolites in both prokaryotic and eukaryotic organisms. PKSs can be grouped into three distinct classes, types I, II, and III, based on enzyme structure, substrate specificity, and catalytic mechanisms. The type III PKS enzymes function as homodimers, and are the only class of PKS that do not require acyl carrier protein. Plant type III PKS enzymes, also known as chalcone synthase (CHS)-like enzymes, are of particular interest due to their functional diversity. In this study, we mined type III *PKS* gene sequences from the genomes of six aquatic algae and 25 land plants (1 bryophyte, 1 lycophyte, 2 basal angiosperms, 16 core eudicots, and 5 monocots). *PKS III* sequences were found relatively conserved in all embryophytes, but not exist in algae. We also examined gene expression patterns by analyzing available transcriptome data, and identified potential *cis*-regulatory elements in upstream sequences. Phylogenetic trees of dicots angiosperms showed that plant type III PKS proteins fall into three clades. Clade A contains CHS/STS-type enzymes coding genes with diverse transcriptional expression patterns and enzymatic functions, while clade B is further divided into subclades b1 and b2, which consist of anther-specific CHS-like enzymes. Differentiation regions, such as amino acids 196-207 between clades A and B, and predicted positive selected sites within α-helixes in late appeared branches of clade A, account for the major diversification in substrate choice and catalytic reaction. The integrity and location of conserved *cis*-elements containing MYB and bHLH binding sites can affect transcription levels. Potential binding sites for transcription factors such as WRKY, SPL, or AP2/EREBP may contribute to tissue- or taxon-specific differences in gene expression. Our data shows that gene duplications and functional diversification of plant type III PKS enzymes played a critical role in the ancient conquest of the land by early plants and angiosperm diversification.

## Introduction

Polyketide synthase (PKS) enzymes play critical roles in bridging primary and secondary metabolism in bacteria, fungi, and plants by catalyzing the sequential condensation of two-carbon acetate units into a growing polyketide chain. PKS enzymes are classified into types I, II, and III based on their structural configurations and catalytic mechanisms. Only the type III PKSs are the essential condensing enzymes that act directly on acyl-CoA substracts in the absence of acyl carrier protein (Hopwood and Sherman, 1990). Type III PKS enzymes in plants, also known as the chalcone synthase (CHS)-like family, synthesize various biologically important components responsible for photoprotection, flower pigmentation, antimicrobial defense, pollen fertility, and the induction of root nodulation (Dhawale et al., 1989; Steyn et al., 2002; Shang et al., 2011). Anti-oxidant and anti-cancer properties of polyketides have attracted considerable attention in pharmaceutical engineering (Lila, 2004; Thomasset et al., 2009).

Chalcone synthase-like family enzymes have acquired multiple activities, and include enzymes such as CHS, stilbene synthase (STS), 2-pyrone synthase (2-PS), bibenzyle synthase (BBS), homoeriodictyol/eriodictyol synthase (HEDS), acridone synthase (ACS), benzophenone synthase (BPS), phlorisovalerophenone synthase (VPS), coumaroyl triacetic acid synthase (CTAS), benzalacetone synthase (BAS), *C*-methylchalcone synthase (PstrCHS2), biphenyl synthase (BIS), stilbenecarboxylate synthase (STCS), pentaketide chromone synthase (PCS), hexaketide synthase (HKS), aloesone synthase (ALS), octaketide synthase (OKS), and anther-specific CHS-like (ASCHSLE) (Austin and Noel, 2003). These enzymes use CoA ester substrates that vary from aliphatic to aromatic, from small molecules to large molecules, and also from polar to non-polar (Flores-Sanchez and Verpoorte, 2009).

The crystalline structures of several plant type III PKS enzymes have been characterized. All of these PKSs function through a homodimer and each monomer possesses an active site (Ferrer et al., 1999). Slight but specific modifications in the active site lead to remarkable functional diversity by influencing substrate selection, the number of polyketide chain extensions, and the mechanism of cyclization reactions. This “steric modulation” model is supported by crystallographic elucidation along with catalytic validation (Austin and Noel, 2003). For example, the Thr197Leu, Gly256Leu and Ser338Ile (numbering as in *Medicago sativa* CHS) substitutions in the CHS amino acid sequence resulting in a complete transformation to a 2-PS enzyme (Jez et al., 2000). The Ser132Thr, Ala133Ser and Val265Phe substitutions are enough to change ACS to CHS (Lukačin et al., 2001). The diketide forming activity of *Rheum palmatum* BAS is attributed to Phe215Leu substitution (Abe et al., 2007). In addition, a single change of His to Glu at position 166 alters the substrate preference of AhSTS from *p*-coumaroyl-CoA to cinnamoyl-CoA (Schröder and Schröder, 1992). But current efforts in CHS and STS conversion only partially alter the catalytic reactions through active sites or their geometry (Tropf et al., 1995; Suh et al., 2000). More evidence is needed for these CHS and STS enzymes, even with the help of the crystalline structures of STS (Shomura et al., 2005).

Soon after the *CHS* gene was cloned from *Petroselinum crispum* (Kreuzaler et al., 1979), tissue-specific and environmentally sensitive expression of *CHS* was found to be widespread in plants. The expression of *PhCHS-A* and *J* in *Petunia hybrida* are restricted to the flower with high levels, while *PhCHS-B* and *G* transcripts are induced by UV light in vegetative tissues (Koes et al., 1989). Except for family members that are expressed in the leaves and flowers, several *M. sativa CHS* genes are preferentially expressed in roots and nodules, and can be induced by pathogen inoculation (Dhawale et al., 1989; Junghans et al., 1993). In *Ipomoea purpurea, IpCHS-D* and *E* are predominantly expressed in the flower limb and tube, while *IpCHS-A/B/C* are expressed at very low levels (Johzuka-Hisatomi et al., 1999; Durbin et al., 2000). Among two characterized members of the *Antirrhinum majus CHS-like* gene family, *AmCHS1* mRNA specifically accumulates in the petal, whereas *AmCHS2* expression is negligible in the petal and other organs (Sommer and Saedler, 1986; Hatayama et al., 2006). In *Gerbera hybrida, GhCHS1* and *GhCHS3* are specifically expressed in the pappus, *GhCHS4* expression is dominant in petals and red vegetative tissues, and *Gh2PS1* (*GhCHS2*) is universally expressed in all tissues (Helariutta et al., 1996; Deng et al., 2013). The MYB-bHLH-WDR ternary transcriptional activation complex was proposed to regulate *CHS* transcription, and this has been confirmed in a number of species (Xu et al., 2015; Zhu et al., 2015). In addition, a group of anther-specific *CHS-like* genes highly express themselves in uninuclear microspores and the tapetum. They exhibited significant differences in amino acid sequences compared to other CHS-like family members (Atanassov et al., 1998). However, the regulatory mechanisms for these *CHS-like* genes are not well studied.

We have observed that there is great diversity in PKS III enzyme gene coding sequences or in the regulatory elements. Amino acids in the active site provide substrate- and product-specificity to PKSs *in vivo* or *in vitro*. Also, *in vivo* expression levels of genes in different cell types constitute another dimension of specificity. Similar to key effective sites in the protein, potential *cis*-elements in regulatory regions can undergo equally dramatic changes and stabilization, corresponding to developmental or environmental regulation (Dey et al., 2015). However, gain-of-function and loss-of-function changes in protein coding sequences or regulatory regions always occur alternatively during evolution, a combination that offers the most fitness for the populations that will be selected by nature (Castillo-Davis et al., 2004; Moore and Purugganan, 2005). When considering the PKS III family, product categories determined by enzyme structure/function and relative expression levels determined by regulatory elements all have specific biological significance. It will be meaningful to dissect out the evolutionary features out of these interlaced aspects by answering questions such as: (1) What diversification patterns did the PKS III family follow? (2) How is the diversification of enzyme sequences and regulatory elements connected with each other?

Fortunately, the public omics database, along with experimental validations, provide convenient source of molecular genetic data. Here we performed genome-wide searches for type III *PKS* gene sequences from 25 land plant species, and collected tissue-specific transcriptional abundance information from RNA-seq or MicroArray transcriptomes of core eudicots, in order to investigate the comprehensive evolutionary pattern of this family from enzyme sequences, regulatory elements, and the combination of the two.

## Materials and Methods

### Identification of *PKS III* Homologous Genes in Plants

The genome sequences of 31 plant species were downloaded from several genomics data portals, i.e., Phytozome^1^ (Goodstein et al., 2011), Ensembl Plants^2^ (Kersey et al., 2014), TAIR^3^ (Lamesch et al., 2012), BRAD^4^ (Cheng et al., 2011), and CuGenDB^5^ (full list, **Supplementary Table S1**). Nucleotide searches were performed using BLAST (Altschul et al., 1990) to identify *PKS III* homologs against genome reference sequences or CDS sequences, using *PKS III* sequences from the literatures (Austin and Noel, 2003; Flores-Sanchez and Verpoorte, 2009) as queries. The threshold was set to an *E*-value ≤ 1E-5. A *PKS* gene was preliminary determined if it was found in both CDS and the corresponding genome sequence locus. Hits found only in genome sequences but not in CDS files were defined as “fragment” loci (shown in **Supplementary Figure S1**). Any *PKS* gene with frame shifts, premature termination codons, or low coverage (the alignment length less than 300 bp) was also defined as a “fragment.” Chromosomal locus of PKS genes and fragments were shown in **Supplementary Figure S1**.

In addition, the redundant sequences in four species, *Physcomitrella patens, Vitis vinifera, Glycine max*, and *M. truncatula*, were filtered by using cd-hit program (Li and Godzik, 2006) (threshold: 0.95 for the former two and 0.98 for the latter two). The remaining gene sequences with correct and complete open reading frames (ORFs) were used to construct the phylogenetic trees and estimate expression levels. A list of all sequences is in **Supplementary Table S2**.

### Phylogenetic Analysis of PKS III Proteins

Predicted amino-acid sequences translated from protein coding nucleotide sequences were aligned with MAFFT (Katoh et al., 2002), then transformed into corresponding codon sequences using PAL2NAL (Suyama et al., 2006). A test of substitution saturation was performed in DAMBE (Xia and Xie, 2001). The best-fit amino acid substitution model (JTT+G) was selected by MEGA (Tamura et al., 2011). Maximum likelihood (ML) analyses were performed in RAxML (Stamatakis et al., 2008) with 1000 bootstrap replications. A Bayesian inference (BI) tree was conducted in MrBayes (Huelsenbeck and Ronquist, 2001). Two independent MCMC runs, each with four chains (three hot, one cold) were run simultaneously starting from a random tree, with sampling stopping when the convergence diagnostic falls below 0.01. The first 25% samples were discarded as burnin, and the remaining trees were used to construct the 50% majority-rule consensus tree.

### Selection Analysis

To detect the changes in evolutionary rates and signatures of positive selection, we analyzed the alignments of codon sequences and the ML tree under a ML framework using CODEML program in PAML 4.8 (Yang, 2007). The one-ratio model assumes the same ω (ω = dN/dS; where dN is the non-synonymous substitution rate and dS is the synonymous substitution rate) for all branches. The two-ratio model assumes a foreground ω parameter for each appointed branch and a background ω for all other branches (Yang, 1998). Models were compared using likelihood ratio tests (LRTs) of the log likelihood (InL). 2| ΔlnL| values between models prepared and degrees of freedom were used in a *chi*-square test with a significance threshold of *P* < 0.01. Because two-ratio models showed that the ω-values for several branches were significantly different from the one-ratio models, we further used branch-site model A to test for sites that were potentially under positive selection on the branch. The model assumes four classes of sites. The first two sites have ω0 (0 < ω0 < 1) and ω1 (ω1 = 1) along all lineages in the phylogeny, whereas the third and fourth have ω2 along the appointed branch, but ω0 and ω1 along other background branches. The branch-site model A was compared with the null model and with the nearly neutral model (M1) (Yang and Nielsen, 2002). Results from PAML are given in **Supplementary Table S3**. Ancestral state reconstruction analysis was performed in MEGA (Tamura et al., 2011).

### Tissue Specific Expression Levels

Gene expression data for different tissues (root, stem, leaf, flower, and fruit) were obtained from public databases (**Supplementary Table S1**). Microarray data was normalized using the RMA method (Irizarry et al., 2003). RNA-seq read data was first filtered using the NGSQCtoolkit, then mapped to reference genome sequences with TopHat (Trapnell et al., 2009). FPKM values were calculated and normalized with the Cuffquant and Cuffnorm pipeline in Cuﬄinks (Trapnell et al., 2013). All values were Log2-transformed.

In order to compare transcript abundance between species, expression levels were transformed to a range of 0–1 within each species by formula: (target value-minimum value)/(maximum value-minimum value). Figures were generated by *ggplot2* package in R (Wickham, 2009). Expression values were listed in **Supplementary Table S4**.

### Conserved Motif Analysis

Upstream sequences of *PK*S III genes from -2000 to the initiation codon were obtained by using BioMarts (Kinsella et al., 2011) or PERL scripts. These sequences were first submitted to PLACE (Higo et al., 1999) or PlantCARE (Lescot et al., 2002) for searching the annotated motifs. Motifs gathered to regions from -1000 upstream to the initiation codon. Then, the MEME suite (Bailey et al., 2009) was used to analyze conserved motifs among all upstream sequences, or among sequences from each lineage *de novo*. We ran the MEME program under the “anr” (any number of repetitions) mode to find motifs exhaustively, and then used TOMTOM (program in MEME suite) to compare motifs found in different lineages. By doing this, a motif distributed around -300 to -100 bp away from the ATG codon was found to be conserved in the majority of upstream sequences. Again using MEME, sequences from -1,000 to the ATG sequences were executed under the “zoops” (zero or one occurrence per sequence) mode, among all upstream sequences, or among sequences from each lineage. Motifs identified *de novo* were annotated by GOMO (program in MEME suite), or submitted to PLACE (Higo et al., 1999) or PlantCARE (Lescot et al., 2002) to annotate.

## Results

### Result 1 Genome-Wide Distribution and Copy Number Variation in *PKS III* Gene Family: Genes from Moss to Flowering Plants

**FIGURE 1:**
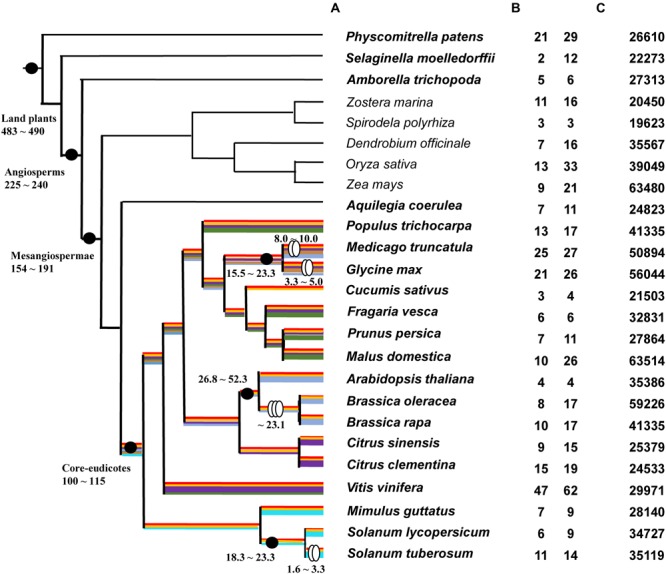
**Phylogenic relationships of 25 land plants and *PKS III* gene number variations. (A)**: Phylogeny of 25 land plant species used in this study. Black dots indicate the important evolutionary nodes. Two ovals indicate diploidizations and three ovals indicate triploidizations. Time point (MYA) estimations are based on previous works or reviews (Blanc and Wolfe, 2004; Chaw et al., 2004; Sanderson et al., 2004; Adams and Wendel, 2005; Soltis et al., 2008; Kagale et al., 2014; Zeng et al., 2014). Highlighted color lines on branches show predicted diversification process according to branch clusters in **Figure 2**. **(B)** *PKS III* gene copy number variations (CNVs) in each genome; the left-hand column is the number of genes with complete open reading frames (ORFs), and the right-hand column is the total number of BLAST hits. **(C)** Total number of genes in each genome.

Thirty-one plant species with whole-genome sequences were chose. Amongst these are six algae (*Micromonas pusilla, Coccomyxa subellipsoidea, Ostreococcus lucimarinus, Volvox carteri, Chlamydomonas reinhardtii*, and *Klebsormidium flaccidum*), one moss (*P. patens*), one lycophyte (*Selaginella moellendorffii*), two basal angiosperms (*Amborella trichopoda* and *Aquilegia coerulea*), 16 core eudicots (*V. vinifera, Citrus sinensis, C. clementine, Arabidopsis thaliana, Brassica oleracea, B. rapa, Populus trichocarpa, M. truncatula, G. max, Cucumis sativus, Fragaria vesca, Prunus persica, Malus domestica, Mimulus guttatus, Solanum lycopersicum*, and *S. tuberosum*), and five monocots (*Zostera marina, Spirodela polyrhiza, Dendrobium officinale, Oryza sativa*, and *Zea mays*) (**Supplementary Table S1**). Whole-genome sequences and putative gene sequences were both used in BLAST searches to identify type III *PKS* genes. The BLASTN with threshold *E*-value = 1E-5 was not available to identify any *PKS III* gene from all six algae genome data. In contrast, all of the 25 land plant species gave positive hits. A phylogenetic species tree of these land plants is shown based on the APG III and updates (Angiosperm Phylogeny Group; Moore et al., 2011) (**Figure 1A**). The copy number variation (CNV) of *PKS* genes with complete ORF are given in **Figure 1B** left and right columns. The total number of putative genes in each genome is also shown (**Figure 1C**).

The numbers of complete ORFs and fragments revealed that *PKS III* family genes occupy a very small proportion of genomes, and their numbers expand in the genomes of moss, grape, and two bean species. Association with recent whole-genome duplication events occurring at the family or genus levels; for example, in the Brassicaceae, after speciation from the common ancestor of *Arabidopsis* and *Brassica*, the ancestor of *B. rapa* and *B. oleracea* experienced a triploidization event. Whole-genome polyploidization resulted in an increase in chromosome and gene numbers; although many species subsequently experienced a reduction in chromosome numbers and gene loss (Cheng et al., 2014; Kagale et al., 2014), the total number of genes increased. We compared species branches before and after triploidization, and found that the rate of increase for this gene family was slightly higher than the average (4/8, 4/10 vs. 35386/59220, 35386/41019). In Solanaceae, the ancestor of *S. tuberosum* experienced diploidization, but the ancestor of *S. lycopersicum* did not (De Bodt et al., 2005; Maere et al., 2005), and the retention rate was similar (6/11 vs. 34727/35119) (**Figures 1B,C**).

When surveying the chromosomal distributions of PKS genes or homologous fragments, we found that most *PKS* loci show scattered chromosomal distributions, while some copies exhibit tandem repeats. Family members embedded within small chromosomal regions always showed higher similarity (95–100%) than other copies (<95%; **Supplementary Table S2**), e.g., *P. patens*: chr2 (24507741-24814900), chr19 (2508254-3629901), *V. vinifera*: chr10 (14216111-14306520), chr16 (16238965-16711898), *M. truncatula*: chr1 (44127878-44142083), chr7 (5283754-5315993), and *G. max*: chr8 (8384741-8519303) (full list, **Supplementary Figure S1**). These highly similar sequences were excluded from our following analyses since they brought redundant calculations.

**FIGURE 2:**
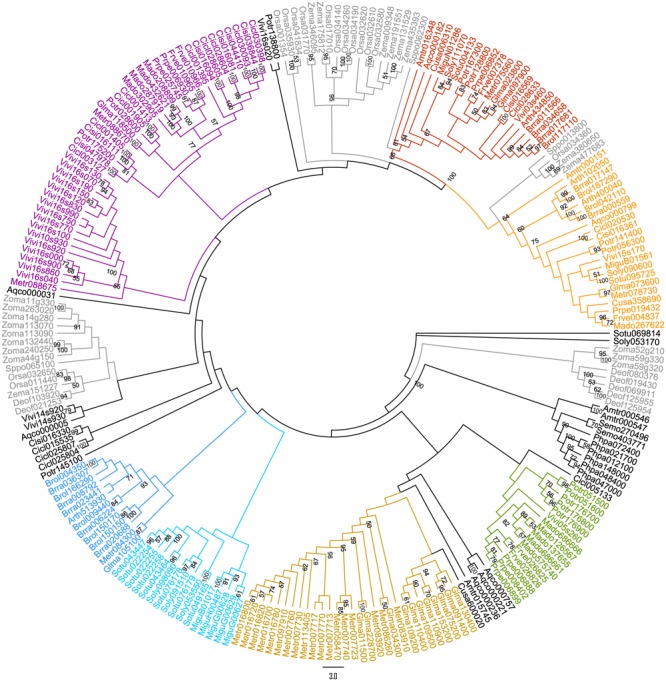
**Maximum likelihood (ML) tree of 234 protein sequences (**Supplementary Table S2**) from 25 land plants (one bryophyte, one lycophyte, two basal angiosperms, 16 core eudicots, and 5 monocots)**.

A preliminary ML tree (**Figure 2**), which was constructed using 234 PKS III protein sequences with complete function structure from all 25 land plants. The PKS III ML tree mirrored the species phylogenetic relationships between these taxa, as the branches of Leguminosae, Rosaceae, Scrophulariaceae-Solanaceae, and Gramineae were identified. Associating with the highly determined evolutionary relationships of these species, we speculated the expansion and diversification history of PKS III family in the lineage of core eudicots (**Figure 1A**). Diversifications mainly happened at the divergence points of Superrosides and Superasterids, and Fabidae and Malvidae, and fixed at the family level during the specification and gene loss events. The last two branches of the tree, composed of sequences covering all of the angiosperm taxa, revealed extreme conservation of this kind of PKS III enzyme. It would be interesting to further analyze the protein structures and expression patterns of genes in these branches. The monocots are estimated with an early divergence time from basal angiosperms than the core eudicot lineage (Zeng et al., 2014), which evolved along a different way in many tissues comparing to eudicots. Sequences from monocots formed four branches in this gene tree, but a clear gene evolutionary history cannot be inferred by this phylogenetic pattern. Whole-genome sequencing programs of monocots were not as adequate as core eudicots at the moment, so we will exclude monocot species in the next analysis.

### Result 2 Phylogenetic Relationship and Protein Sequence Diversification within PKS III Family

In order to understand diversification patterns of PKS III proteins, we focused on core eudicot lineage sequences. A number of 226 sequences were collected, including 190 complete PKS III ORFs from projected sampled species (**Figure 1**, bold letter species) with monocots removed, and 36 functional validated reference sequences in previous work (full list, footnote of **Figure 4**).

**FIGURE 3:**
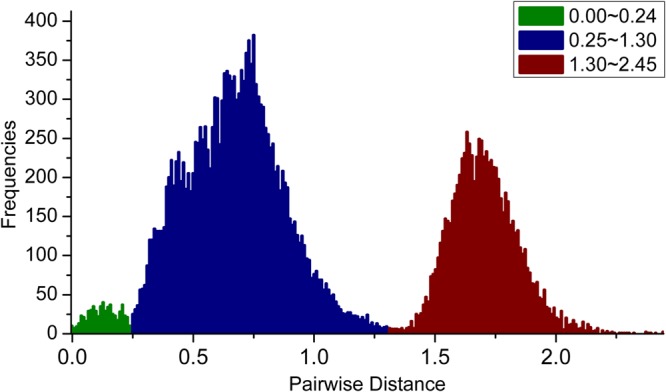
**Frequency distribution of pairwise genetic distances (transitions and transversions) for *PKS III* genes.** Three peaks, with genetic distances ranging from 0–0.25, 0.25–1.3, and 1.3–2.5, are shown in green, blue, and red, respectively.

We first calculated the pairwise distances (transitions and transversions) for these *PKS III* gene sequences, and calculate their frequency distribution (**Figure 3**). As a result, three peaks ranging from 0–0.25, 0.25–1.3, and 1.3–2.5 are displayed separately. Values forming the 0–0.25 peak originated mainly from clustered genes and homologous genes of very closely related species. The sequence relationships which forming the values in the latter two peaks could be distinguished by phylogenetic analysis using protein sequences.

**FIGURE 4:**
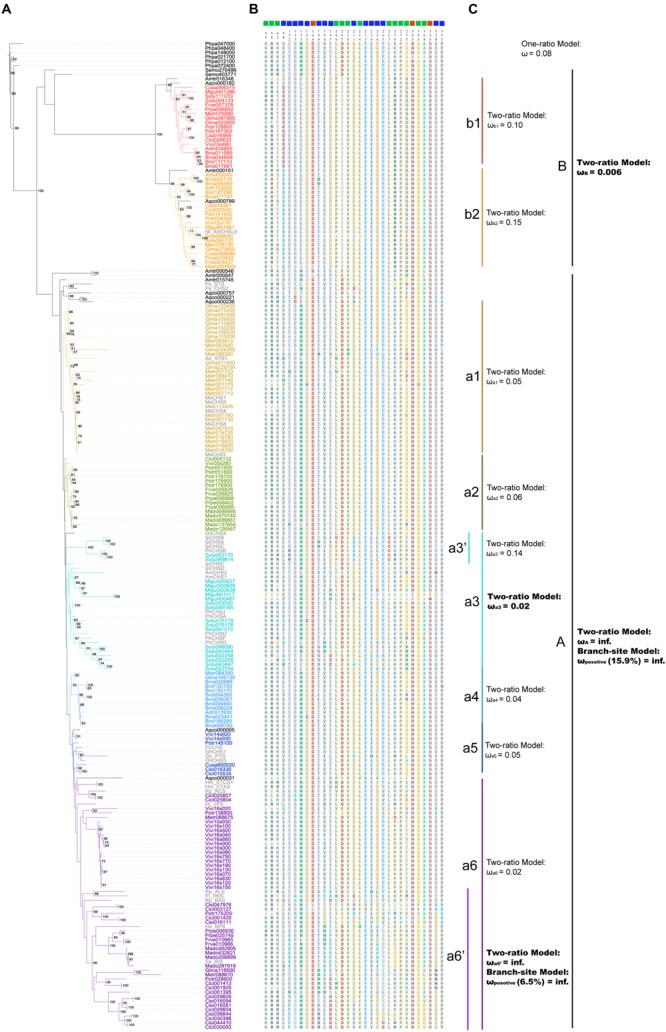
**Maximum likelihood tree showing evolutionary relationships for basal angiosperm and core eudicots PKS III proteins, with corresponding enzyme active sites and estimates of selective pressure. (A)** ML tree of plant PKS III proteins. The protein names in color are 190 sequences from 20 species (bold letter species in **Figure 1**). Thirty-six names in gray are proteins translated from nucleotide sequences mentioned in previous works. List as follows: *Petroselinum crispum PcCHS* (V01538.1) (Reimold et al., 1983), *Antirrhinum majus AmCHS1* (X03710.1), *AmCHS2* (AB219808.1) (Sommer and Saedler, 1986; Hatayama et al., 2006), *Gerbera hybrid GhCHS1* (Z38096.1), *Gh_PYS* (Z38097.2), *GhCHS3* (Z38098.1), *GhCHS4* (AM906210.1) (Helariutta et al., 1996; Deng et al., 2013), *Medicago sativa MsCHS1* (L02901.1), *MsCHS2* (L02902.1), *MsCHS4* (L02903.1), *MsCHS8* (L02904.1), *MsCHS9* (L02905.1) (Junghans et al., 1993), *Ipomoea purpurea IpCHSA* (U84905.1), *IpCHSB* (U15947.1), *IpCHSC* (U15949.1), *IpCHSD* (AF358657.1), *IpCHSE* (AB027534.1) (Durbin et al., 2000), *Petunia hybrid PhCHSA* (X14591.1), *PhCHSB* (X14592.1), *PhCHSD* (X14593.1), *PhCHSF* (X14594.1), *PhCHSG* (X14595.1), *PhCHSJ* (X14597.1) (Koes et al., 1989). And sequences follows (Austin and Noel, 2003; Flores-Sanchez and Verpoorte, 2009): *Arachis hypogaea Ah_STS1* (AB027606.1), *Pinus sylvestris Ps_STS* (X60753.1), *Pinus strobus Ps_CHS2* (AJ002156.1), *Ruta graveolens Rg_ACS* (AJ297786.2), *Hypericum androsaemum Ha_BPS* (AF352395.1), *Humulus lupulus Hl_VPS* (AB047593.2), *Hydrangea macrophylla Hm_CTAS* (AB011468.1), *Hm_STCS1* (AF456445.1), *Rheum palmatum Rp_BAS* (AF326911.1), *Rp_ALS* (AY517486.1), *Nicotiana sylvestris Nt_ASCHSLE* (Y14507.1), *Plumbago indica Pi_HKS* (AB259100.1), *Sorbus aucuparia Sa_BIS* (DQ286036.1). **(B)** Enzyme active sites predicted from crystal structure and functional confirmation. **(C)** Selective pressure tests results from PAML.

**FIGURE 5:**
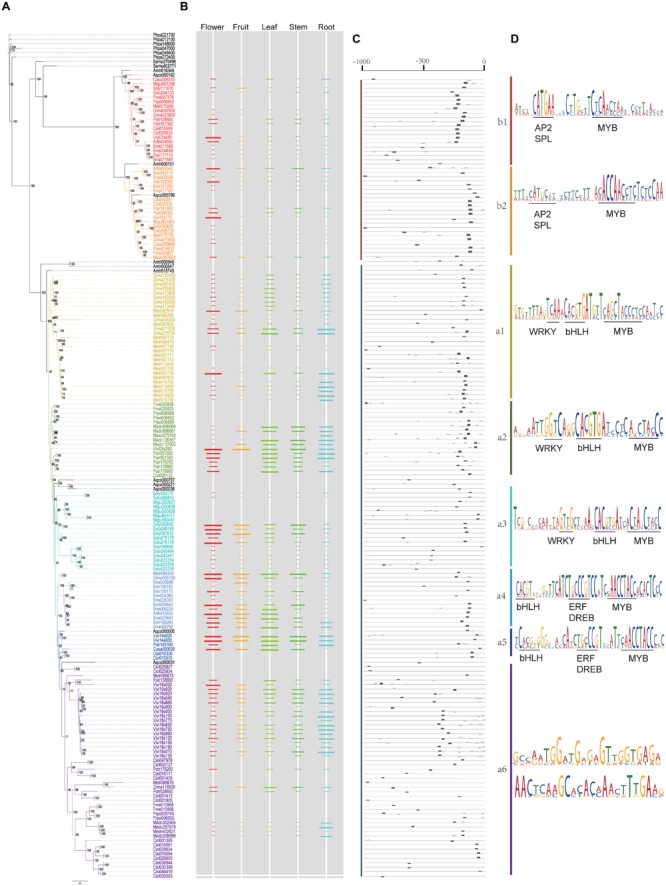
**Bayesian inference tree of 190 plant PKS III proteins from 20 species, with corresponding gene expression levels and conserved *cis*-elements predicted. (A)** BI tree of plant PKS III proteins. **(B)** Gene expression levels were normalized from tissue-specific transcriptomes. The length of the colored bars indicated the relative expression level within a genome. Spaces without colored bar represent missing value. **(C)** Locations of conserved sequences. **(D)** Conserved sequences and *cis*-elements estimated.

Polyketide synthases III protein sequences were used to construct the ML (**Figure 4A**) and BI trees (**Figure 5A**). When constructing the ML tree, PKS III sequences with crystal structures and enzymatic activity verification summarized in reviews (Austin and Noel, 2003; Flores-Sanchez and Verpoorte, 2009) and CHS-like sequences from previous studies (see Introduction and **Figure 4A**) were served as references. The main phylogenetic patterns between the ML tree and BI tree were highly consistent. The PKS III proteins of seed plants formed into two clades, designated A and B. Clade B can be further divided into subclades b1 and b2. The tobacco anther-specific CHS-like protein Nt_ASCHSLE is clustered in clade b2, suggesting a subgroup with distinctive function from other known members. Clade A is comprised of a large number of PKSs, embracing almost all types of reference PKS enzymes except Nt_ASCHSLE. PKS III sequences from closely related taxa grouped together, comprising diverse branches in clade A, designated a1 to a6. When using exemplary PKS III sequences as references (colored gray in **Figure 4A**), CHS/STS occupied more basal positions in clade A. Branch a6 contained more diverse types of PKS III enzymes that exhibited key amino acid residue substitutions in the active sites.

We show the amino acids of the catalytic triad (red squares), coenzyme A binding site (green squares), and functional diversity (blue squares) sites for 226 sequences in the ML tree, based on the annotated sequence and secondary structure of alfalfa CHS (MsCHS2 in the tree; **Figure 4B**). Among the three types of residues, the catalytic triad was the most conserved, while the other two showed slight diversification in different phylogenetic groups. If we only consider the conserved amino acid residues of the active site, clade B shows relatively large differences when compared to moss and fern sequences and clade A. We combined the active site residues with whole sequence alignments, and although large differences were present in clade B, the catalytic triad residues Cys164, His303, and Asn336, and “gatekeeper” residues Phe215 and Phe265 of the core chemical machinery were conserved. Significant differences were found in two sections in β4 and β6, 98–138 and 196–207, both functional diversification hot-spot regions of PKS III family enzymes. The alfalfa CHS Met137 and Pro138 residues are regarded as the contact point of two monomers (Jez et al., 2000), suggesting that dimer formation related components might play an important role in function diversification of clade B enzymes. Differences in residues 98 and 196–207 may be associated with CoA substrate alteration.

**FIGURE 6:**
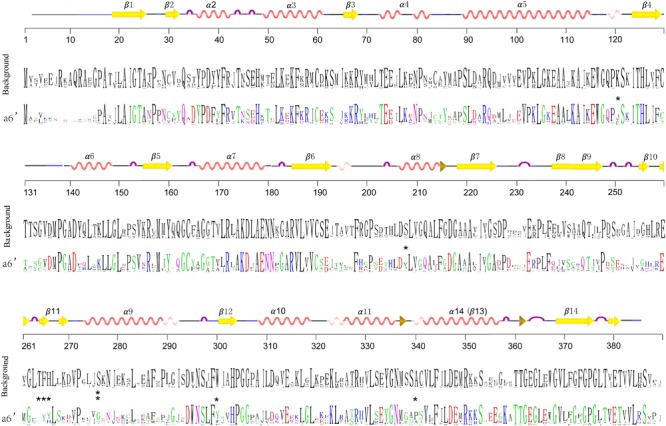
**Sequence frequency of amino acid residues of branch a6’ (colored) and other branches (black).** Generated by WebLogo (http://weblogo.berkeley.edu/logo.cgi) (Crooks et al., 2004). The amino acid positions are numbered relative to alfalfa CHS2. Protein secondary structures of protein were predicted based on the three dimensional structure of alfalfa CHS2 (1BI5). Stars show the significant positive sites estimated by the Branch-sites models.

In order to detect changes in selection pressure, we performed one-ratio, two-ratio and branch-site models in each branch using PAML (**Figure 4C**; **Supplementary Table S3**). Selective pressures in clades A and B in the two-ratio models were all significantly different from the one-ratio model. Positive selection was detected in clade A, while clade B members were under more constrained purifying selection (0.006). In clade B, selective pressure values for the two subclades b1 (0.10) and b2 (0.15) were relatively similar. Divergence in regions of clade B enzymes also show further divergence in subclades b1 and b2, such as active site residues 98 and 196. In clade A, branch-site models against each branch revealed that positive selection was mainly in branch a6’. The positive sites were estimated to be 121 K→I, 208 S→V, 276 S→G, 300 W→Y, 340 A→P, located in α-helix type secondary structures, and 264 T→E, 265 F→Y, 266 H→Y, located in β-turn number 11 (**Figure 6**). The evolutionary process in branch a6’ sequences at these amino acid sites through ancestral state reconstruction analysis are also shown (**Supplementary Figure S2**).

### Result 3 Transcriptional Expression Levels of *PKS III* Genes Were Related to *Cis*-Elements

Polyketide synthases III enzymes participate in secondary metabolic processes that are important to plant development and defense. How and when the various genes are expressed is an important aspect of functional diversification. Currently, large amounts of transcriptome data have been submitted to public databases, with sampling from various tissues and growth conditions. Tissue samples taken under natural growth conditions can allow for direct comparison between all species after data normalization. Therefore, we collected gene expression data from different tissues, including root, stem, leaf, flower, and fruit. We also included some specialized organs, such as tendrils of cucumber and nodules of soybeans. Because the expression levels of *PKS III* genes were highly correlated with certain tissues, we found that five main tissues could adequately represent their gene expression profiles. We found ten angiosperm species with such tissue-specific data sets. After technical normalization to eliminate experimental error caused by sampling and sequencing, Log2-transformed expression values were then 0–1 transformed within each species (see Materials and Methods).

The relative gene expression levels for the five tissues are shown adjacent to the phylogenetic tree in **Figure 5B**. Although they are from different species and data types, gene expression levels displayed regular features along phylogenetic lines. Most genes in clade B showed expression at low abundance, but some showed increased expression in floral organs, which were most obvious in *A. thaliana* and *V. vinifera*. The increased expression in flowers might come from the anthers, as confirmed experimentally in other work (Atanassov et al., 1998). Genes in the branch a1 of clade A often displayed a root-specific expression pattern, with a few exceptions that are expressed in flowers. Genes in a2 are generally expressed in vegetative tissues, while a3 and a4 genes are universally expressed in above-ground tissues. In branch a5 genes, transcript abundance seemed equivalent among the five tissues. Branch a6 genes were similar to branch a5, but with slightly lower levels.

DNA sequences -1000 bp upstream (5′) of the ATG initiation codon were used to search for conserved motifs and annotated *cis*-elements. When we queried the annotated motif databases PLACE or PLANTCARE, large numbers of *cis*-elements were found, which are annotated as responsive to light (G-box, ATCT-motif, AE-box, ACE, ATCC-motif, GAG-motif, and GARE-motif), hormones (ABRE, ERE, AuxRR-core, GARE-motif, and P-box), and elicitors (EIRE and Box-w1). This matches the general defensive function of the PKS gene family. In general, the numbers of *cis*-elements present in upstream sequences were positively correlated with gene expression levels. However, these *cis*-elements always overlapped with each other, or appeared to be distributed randomly throughout the sequences, making it difficult to find meaningful patterns.

We then attempted to discover conserved motifs by MEME suites, using all of the upstream sequences or within each lineage. We first ran the MEME program using all of the upstream sequences, but no conserved motifs were found. This might be because of the high divergence between clades A and B, so we analyzed each clade separately. As a result, the most conserved motifs in clades A and B were identified. As shown in **Figure 5C**, the most conserved motif of all clades distributed from -300 to -100 bp upstream (5′) of the ATG start codon. Except for the missing values, genes expressed in at least one tissue all had a complete motif that was suitably located (**Figures 5B,C**). As expected, the most conserved motif of clade A genes embraced adjacent MYB and bHLH binding sites, two transcription factors of the MBW transcriptional regulatory complex, which have been experimentally verified in several species (Wang et al., 2013; Zhu et al., 2015). We then analyzed the conserved motif in each sub-clade. According to the phylogenetic tree, the clade A motif changed slightly between the different branches, including MYB and bHLH binding sites and adjacent nucleotides. We searched and annotated them by referring to databases or comprehensive references in plants (Franco-Zorrilla et al., 2014), and found different rules for the sub-clades along the tree (**Figure 5D**). In branches a1, a2, and a3, a WRKY binding element (TTGACY) was located adjacent and 5′ to the end of the bHLH and MYB binding sites, whereas in branches a4 and a5, the bHLH and MYB binding sites were a little farther away from each other, and had a predicted ERF or DREB binding element (containing GCC-box) between them. But in branch a6, the motif containing bHLH and MYB binding sites is not conserved in the sequences, which made it difficult to find more significant information. Another two conserved motifs in branch a6 are given for further verification. On the other hand, the clade B-specific motifs also have a MYB-binding site, with one or more G-boxes in the promoter, but the adjacent element was determined to be a core-GTAC sequence, which is similar to the AP2 binding element or SPL binding element (Franco-Zorrilla et al., 2014). This element could potentially be associated with anther-specific PKS gene expression during floral development.

## Discussion

Polyketide synthase enzymes use products of primary metabolism to synthetize chemical molecules participating in defense and reproduction. The expansion and stabilization of the *PKS III* gene family was important for the history of organismal adaptation and evolution. Early studies deduced the origins of the flavonoid pathway leading by the CHS enzymes through chemical substance analysis in extant species, and the time scale roughly dated back to the formation of the mosses (Stafford, 1991). Our work confirmed this hypothesis at the DNA sequence level by thoroughly analyzing genes in species ranging from alga to angiosperms. We used functionally verified *PKS III* genes from every major group of plants as queries in BLAST searches against algal and land plant genomes using a relaxed search threshold (≤1E-5), and hit scores provided information about relative sequence similarity. BLAST search results and alignments of nucleotide and protein sequences of higher plant PKS III enzymes showed that they were fixed in genomes of early land plants. Possibly due to protection from the effects of direct exposure sunlight, this event laid the foundation for the rapid conquest of the land by plants. Though common in land plants, the large changes on peptide sequences of this type of PKS in evolutionary history reflect further specialization during the ancestors of basal angiosperms. Another function for sporopollenin biosynthesis of PKS enzymes was also proposed and verified. Anther-specific CHS enzymes of higher plants have homologs in *P. patens* genome and still have complementary functions in *Arabidopsis*, even with extremely low identity value (∼30%) in peptide sequence (Koduri et al., 2009; Colpitts et al., 2011). Clades of basal angiosperms, monocots, and core eudicots (highlighted with red and orange in **Figure 2**) indicated a significant conservation of the anther-specific function among all land plants. Unlike the eudicots each of whom had at least one copy belonging to the two clades, the situation of the five monocots seemed complicated. A few Gramineae PKSs exist in the orange clade, while more members constitute another branch close to the two clades. The Orchidaceae plant *D. officinale* does not have the anther-specific *PKS III* genes. Perhapes the function of those genes diverged during the specification of gynandrium. One free floating plant in Alismatales, *S. polyrhiza*, has sequences belonging separately to the red and orange clades. But one submerged plant in Alismatales, *Z. marina*, which has completely adapted to the aquatic habit, does not have complete *PKS III* genes in its genome. This may occur due to the reproduction strategy transition under the water (Olsen et al., 2016). However, more omics data and experiment validations are needed to dissect the gain or loss mechanisms of angiosperms’ aquaticness.

Successive expansions of this multigene family in extant seed plants are thought to have depended on both genome-wide duplications and small scale duplication events. In the ML (**Figure 4A**) and BI (**Figure 5A**) trees of land plant PKS III, basal angiosperms and core eudicots formed two clades, clades A and B. Previous work has distinguished the ASCHSLE group of sequences as a monophyletic clade (Jiang et al., 2008). Our trees showed that clade B further divided into two lineages, subclades b1 and b2. Genes form clade A, subclades b1 and b2 contain sequences from almost all seed plants, including the most basal angiosperm *A. trichopoda*. This pattern suggested that the genes formed in these three clades were originated through two events occurred in the common ancestor of all angiosperms. It has been determined that two ancient genome-wide gene duplications occurred separately, one in the common ancestor of extant seed plants, and the other in the common ancestor of modern angiosperms (Jiao et al., 2011). We speculate that the split between clades A and B and subclades b1 and b2 are very likely to represent these two genome-wide gene duplication events. Except for genome-wide duplications, small-scale duplications also contribute to the expansion of this gene family. We noticed that *PKS III* family members with clustered chromosomal localization contribute largely in the expansion of clade A. By checking the intron numbers, all these cluster-located genes were found having more than one intron. This excluded the possibility of recent retrotransposition duplication events (Zhang, 2003). Therefore, tandem duplication events should be responsible for another group of *PKS III* gene family members.

During the evolutionary process, extant chromosomal distribution and retention bias of PKSs often adapt to their modes of action whatever the duplication patterns are. In clade B, natural selection conserved *PKS III* genes with scattered chromosomal distributions. This may tend to support the “gene balance hypothesis,” as genes with a tendency to interact with each other were less likely to retain in tandem (Freeling, 2009). Associated with the strong purifying selection pressure of clade B genes, plants do not need so many tandemly repeated PKSs for survival as what they did for rRNA or histone genes. However, the functions of PKSs in the formation of pollen exine are essential. Furthermore, the explicit differentiation between clades b1 and b2 may suggest the heterodimer type of interactions for the anther-specific CHS-like genes. In clade A, considering the genes that are highly expressed are embedded within a single cluster, the tandem copies may increase the accumulation of useful products. Although this often led to redundancy, many backup genes were assurances when facing selection pressure. As in *P. patens*, the presence of many *PKS III* gene copies implies a large requirement for defensive chemicals in early land plants. In addition, domesticated plants with this kind of genome arrangement might be artificially selected for traits of agronomic importance, such as isoflavones in the seeds of legumes and tannins in the fruits of grapes.

Following gene duplications, the diversification of seed plant PKS III can be observed in both enzyme structures and gene regulatory elements (Moore and Purugganan, 2005). Nucleotide mutations leading to amino-acid changes accumulated toward changes in enzymatic action. As mentioned in the results section (**Figure 4B**), the significant differences between clades A and B are in β4 and β6 regions, residues 98–138 and 196–207, respectively. Although difference happened at position 137 (M in clade A and L in clade B), the contact point of two monomers, no properties changed at the amino acid level. Diversification of the large region that includes residues 196–207, which is associated with CoA substrate binding, may be fixed in sequences soon after the diversification of the A and B clades. This supports the large differences in substrate choices between the two main clades. Interestingly, in contrast to clades A and b2, the amino acid at site 98 in subclade b1 proteins caused significant changes in polarity and hydrophilic-hydrophobic properties. Cysteine in b1 and alanine in b2 is absolutely conserved, which suggests the most specific substrate preference possible during tapetum formation. This property transition is important and should be the focus of future research. Due to the repeated gene duplications in specific lineages, sequences forming clade A contribute to the majority of the family members. And because of this, functional diversifications in this clade are more taxon-specific. *CHS/STS* genes, whose end-products are flavonoids or polyphenolics such as resveratrol, make up the majority and represent the ancestral status in this clade. This implies that PKSs, especially typical CHS/STS enzymes, persisted in their role in the conquest of land by early land plants. Furthermore, their functions also were extended. Sequence distributions in the phylogenetic trees might imply the acquisition of new functions by shifting several key sites from CHS/STS to many other kinds of PKS III enzymes in some lineages. This is consistent with the fact that changes in only a few amino acids were enough to change the qualitative enzyme functions from 2-PS or ACS to CHS (Jez et al., 2000; Abe et al., 2007). Although current experimental efforts concerning CHS and STS functions only quantitatively exchanged their substrate preferences (Schröder and Schröder, 1992; Suh et al., 2000). Attempts to exchange functions between CHS and STS enzymes requires more investigation. Selective tests using models in PAML reflected positive or negative selection pressures at branches made up of different phylogenetic groups (**Figure 4C**). The youngest branch a6’, composed with genes from the Rosaceae and Rutaceae, is estimated to be under a significant positive selection at given amino acid residues embedded in several α-helixes (121 K→I, 208 S→V, 276 S→G, 300 W→Y, 340 A→P) and β11 (264 T→E, 265 F→Y, 266 H→Y) (**Figures 4C** and **6**). This suggests that these structures contribute more to the functional diversification of PKS III enzymes. Six of the eight positive positions cause changes in polarity, charge, and hydrophilic-hydrophobic properties. These changes are suggested to influence the steric interactions and relative binding of the substrates and elongating molecules inside the catalytic cavity.

In addition to the protein sequences, gene expression patterns and potential *cis*-elements in upstream regulation regions also showed significant diversification. Anther-specific expression CHS-like proteins, which are embedded in clade B, have the highest expression levels in flowers (**Figure 5B**). The presence of a putative AP2 binding element implies that genes in this clade can respond to endogenous floral developmental signals. In different branches within clade A, expression patterns showed various tissue-specific patterns among closely related species with different biological effects. Leguminosae genes in branch a1 had root-specific expression patterns, and may be related to nitrogen fixation. Distribution of *cis*-elements in the different lineages indicated auxiliary *trans*-regulatory factors, except for MYB and bHLH transcription factors (**Figure 5D**). WRKY binding elements are present in genes from branches a1, a2, and a3, and ERF or DREB binding elements in genes in branches a4 and a5. Genes in a2, a3, a4, and a5 are expressed universally in vegetative or other above-ground tissues, suggesting their induction by broad acting hormones or external environmental signals. AP2, ERF, and EREBP are three subfamilies in AP2/EREBP superfamily, which occupies large proportion of plant transcriptional factors, and proteins in this superfamily widely participate in growth and response regulations (Riechmann, 2000). These findings promote the potential upstream regulatory roles and evolutionary relationships of the AP2/EREBP family proteins to the PKS III genes. By significant patterns revealed from conserved sequences, the predicted elements provide candidates for further experimental validation.

Since changes in protein sequence and expression patterns have been found under regular diversification, they appear to be connected (**Figures 4** and **5**). PKS III in the phylogenetic tree can be roughly classified into two types: the proteins Metr007723-Metr058470-Metr007740 and Soly098090-Sotu043464-Sotu043447-Sotu022254-Sotu022255-Sotu022258 in clade A all have properties like longer branch lengths, loss of *cis*-binding sites, and inability to transcript. The other type, which comprises the majority of members with full functionality, sequence accuracy, motif integrity, and a high level of transcript in at least one tissue. It seemed that protein activity and gene expression levels are two synergetic aspects in the evolution of a multigene family. In the case of PKS III enzymes, the three sites (164C, 303H, and 336N) which make up the catalytic triad (**Figure 4B**), and the predicted MYB binding elements -300 to -100 bases upstream of the initiation codon (**Figure 5C**), are extremely conserved in all *PKS III* genes in all species, which implies the critical importance of these components. The stationary combination of the catalytic triad and *cis*-elements are outcomes of a long-term historical evolutionary process. Additionally, duplication events that occurred in the recent ancestors of certain taxa caused gene redundancy within a relatively short period, leading to relaxed selection pressures on some copies. Considering this, slight alterations in sites adjacent to the key region or *cis*-elements can be more flexible. This might be the reason for the formation of lineage specific patterns of the catalytic reaction and tissue-related expression.

Because of the different metabolic flows in various cell types, *in vitro* experiments cannot reflect the true catalytic reactions *in vivo* completely. At the same time, tissue-specific expression patterns can be influenced by *trans*-acting regulatory factors or even differential epigenetic modifications. These factors make it difficult to elucidate the functional diversification of individual family members. However, as a family of structural genes, in which their end effects on phenotypes result from natural selection, sequence features of structural genes are reflections of plant adaptability. We expect that the results from this study will provide a reference for further evolutionary studies or engineering programs in the field of polyketide synthases.

## Author Contributions

RS and LX designed the study. LX collected and analyzed the data and drafted the manuscript. ZZ, ShiZ, ShuZ, FL, and HZ helped to collect data. PL, GL, and YW helped to analyze data and draft the manuscript.

## Conflict of Interest Statement

The authors declare that the research was conducted in the absence of any commercial or financial relationships that could be construed as a potential conflict of interest.

## Footnotes

1 http://www.phytozome.net/

2 http://plants.ensembl.org/index.html

3 http://www.arabidopsis.org/

4 http://brassicadb.org/brad/

5 http://icugi.org/

## Supplementary Material

FIGURE S1 **Chromosomal distribution of predicted polyketide synthases (PKS) III genes and fragments**.

FIGURE S2 **Ancestral state reconstruction of eight positive sites (121, 208, 264, 265, 266, 276, 300, and 340) in clade a6’**.

TABLE S1 **Information on the genomes and transcriptomes used in this study that were downloaded from public databases**.

TABLE S2 **Sequences list of polyketide synthases (PKS) III genes and fragments in 25 land plant genomes.** Gray filled means sequences with incomplete open reading frames (ORFs). Blue filled means sequences deleted as redundancy by cd-hit. Red filled means sequences located within the same gene cluster.

TABLE S3 **Results of one-ratio, two-ratio and branch-site models from PAML**.

TABLE S4 **Expression levels of *PKS III* genes calculated from transcriptomes data**.
