# Supplementary material for: Phylogeny and Expression Analyses Reveal Important Roles for Plant PKS III Family during the Conquest of Land by Plants and Angiosperm Diversification
Source: Front Plant Sci. 2016 Aug 30;7:1312. doi: 10.3389/fpls.2016.01312 (PMC5004622; doi:10.3389/fpls.2016.01312)
Supplement: FIGURE S1 — Chromosomal distribution of predicted polyketide synthases (PKS) III genes and fragments. [file Image_1.PDF]

Supplemental Figure 1

Chromosomal distribution of predicted PKS genes and fragments.

Arabidopsis thaliana

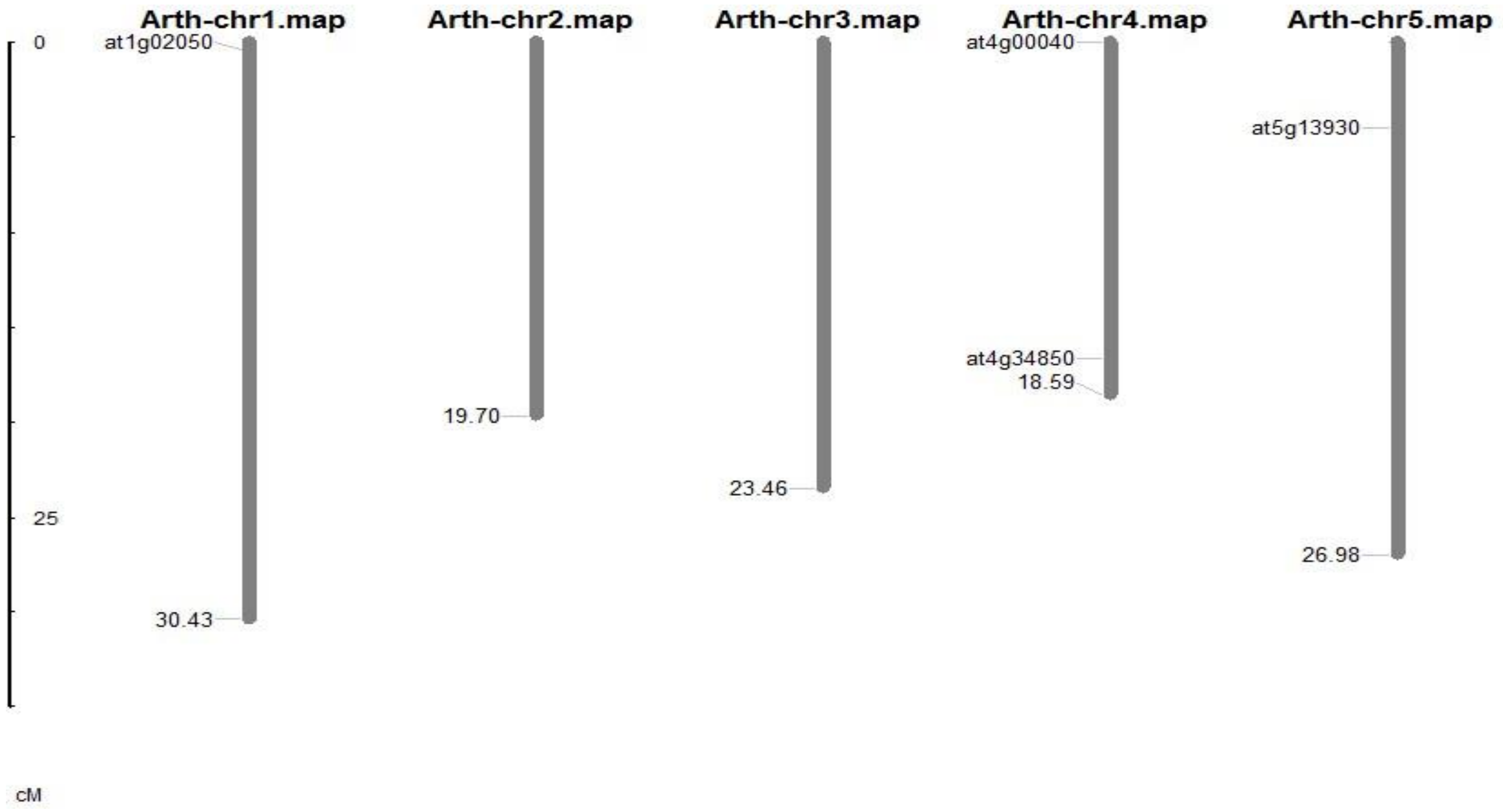

Brassica oleracea

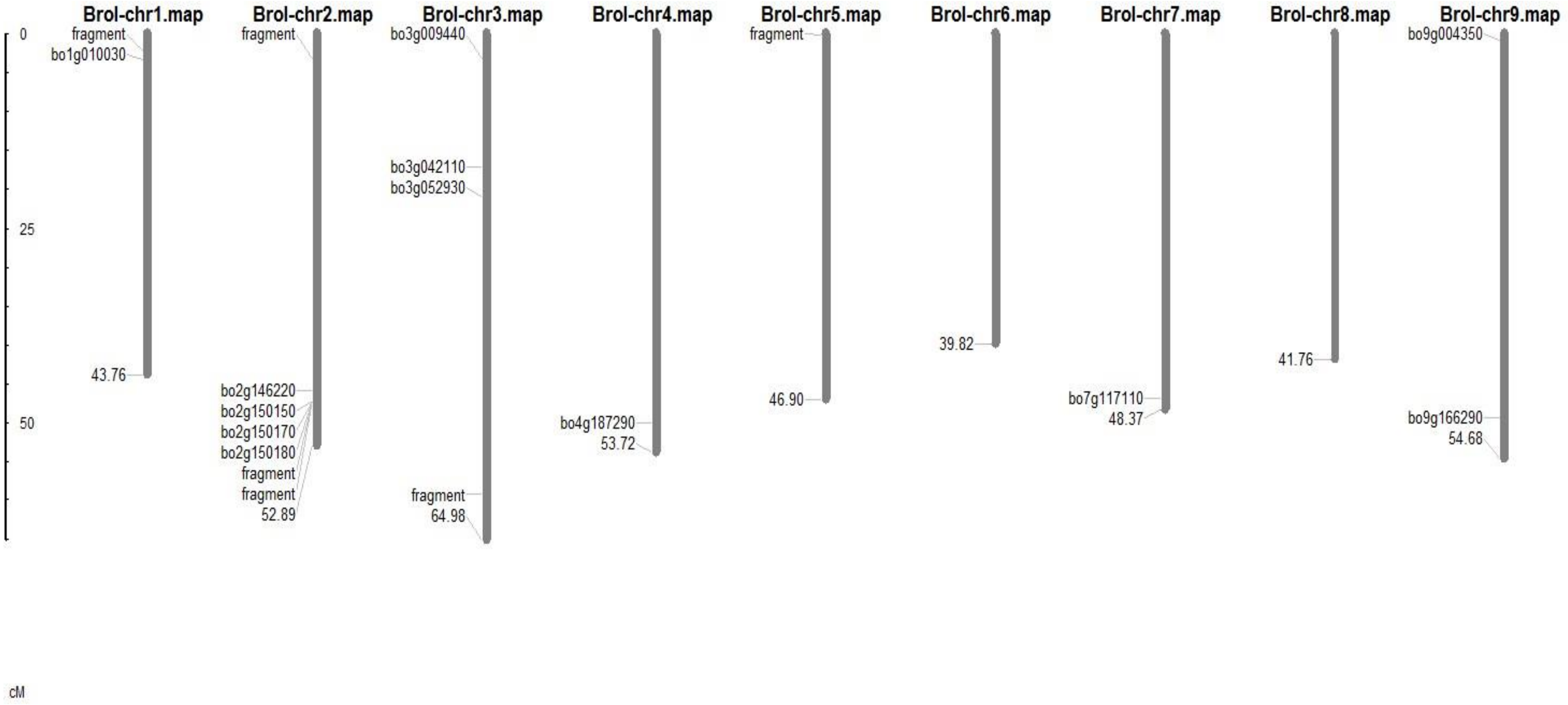

Brassica rapa

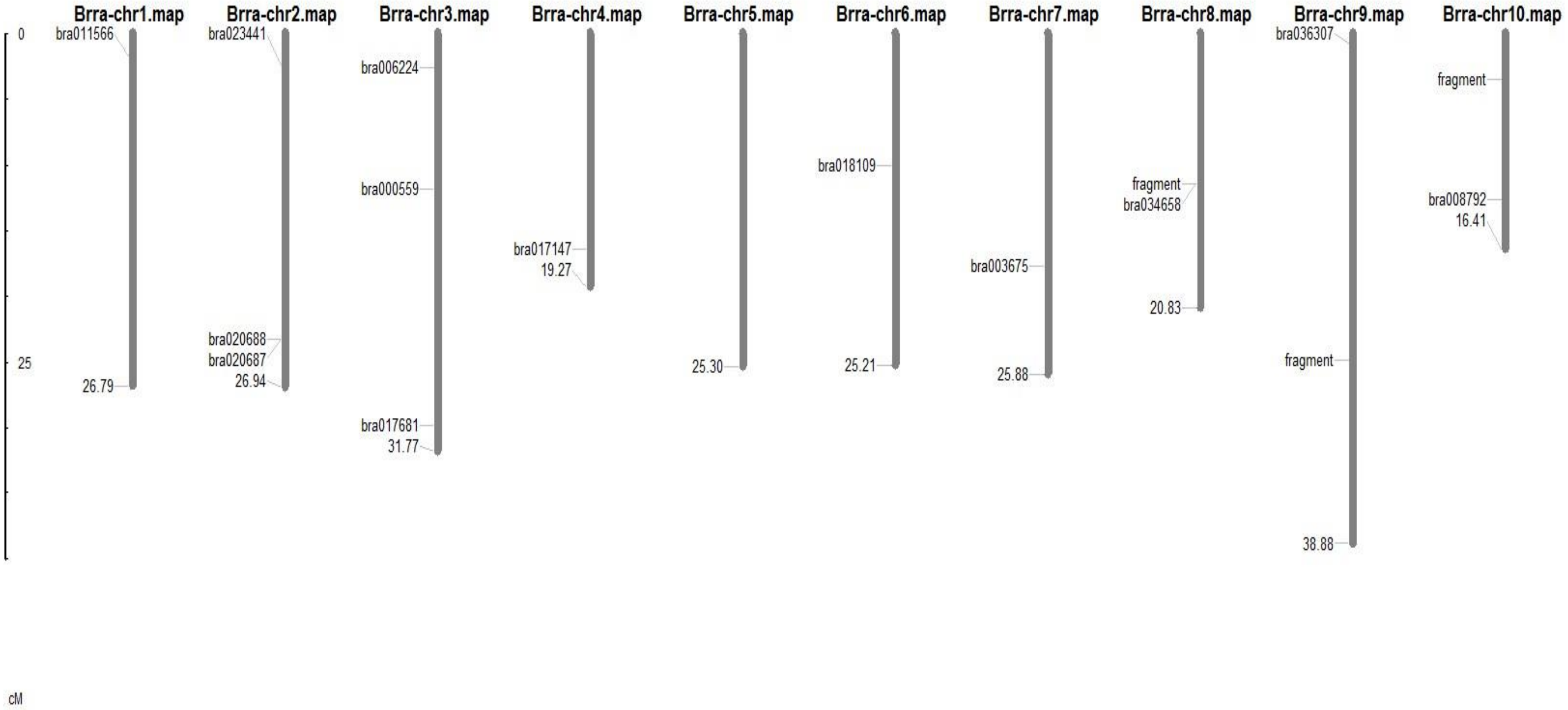

Fragaria vesca

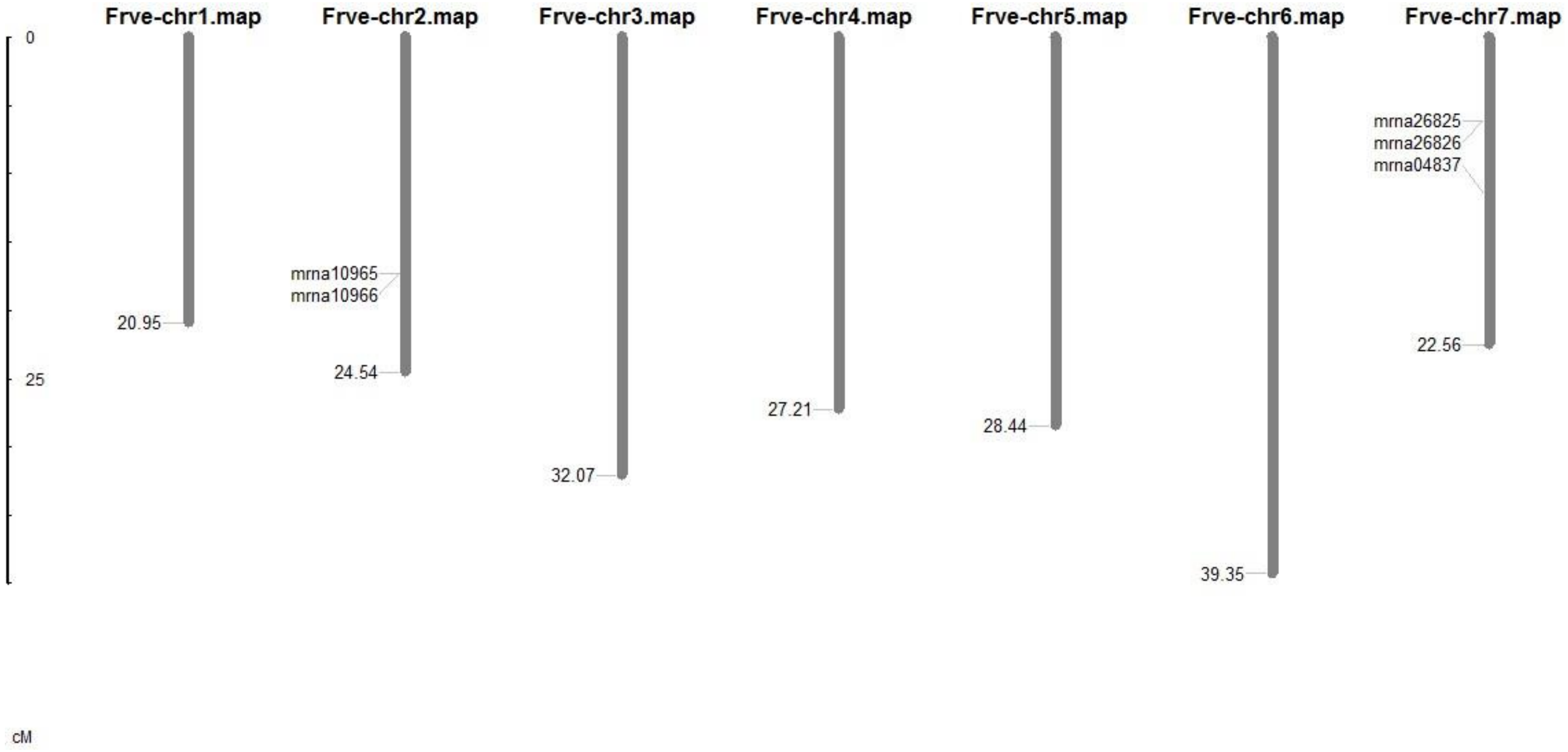

Glycine max

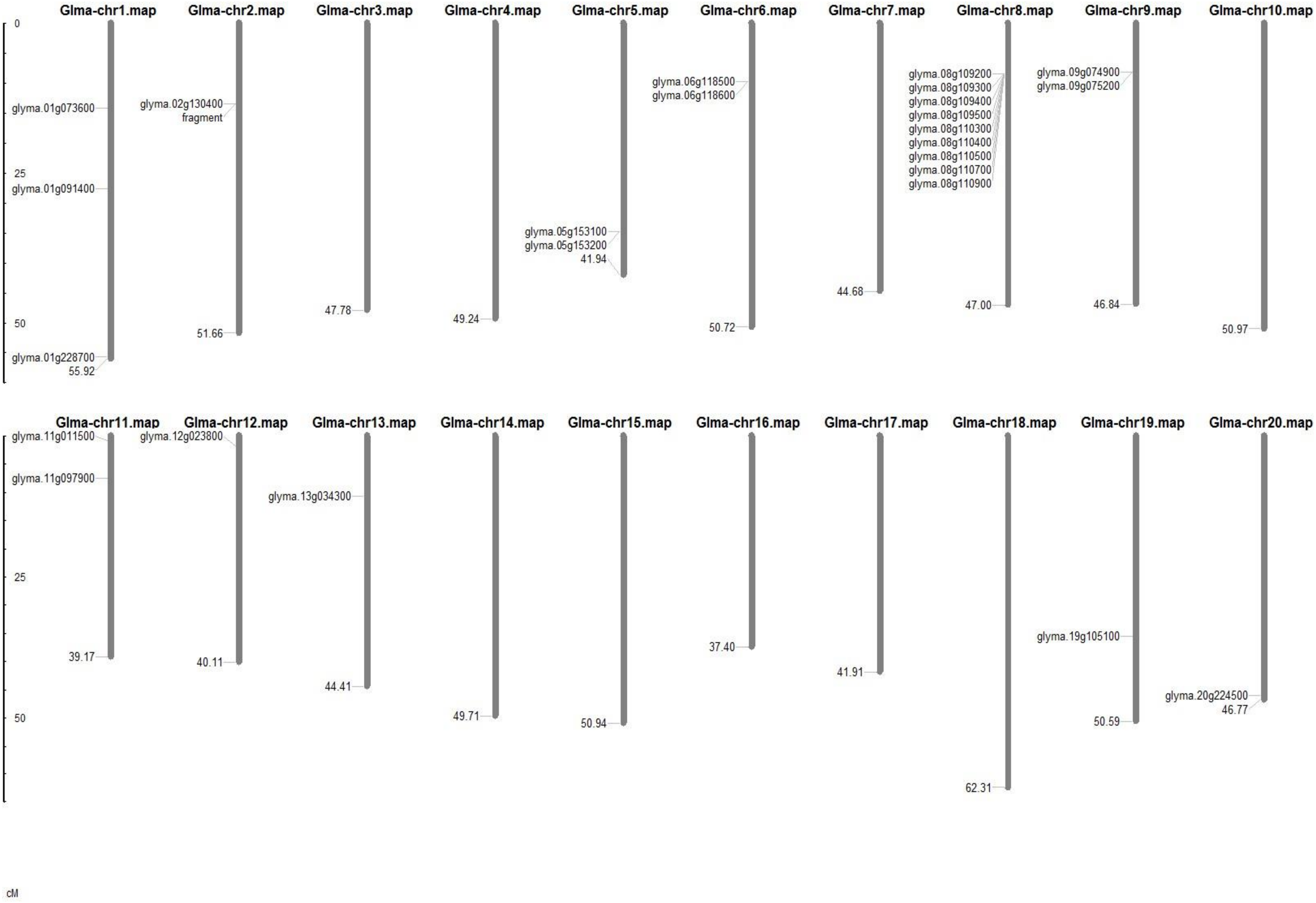
Glma-chr1.map
Glma-chr2.map
Glma-chr3.map
Glma-chr4.map
Glma-chr5.map
Glma-chr6.map
Glma-chr7.map
Glma-chr8.map
Glma-chr9.map
Glma-chr10.map
0
25
50
glyma.01g073600
glyma.01g091400
glyma.01g228700
55.92
glyma.02g130400
fragment
51.66
47.78
49.24
glyma.05g153100
glyma.05g153200
41.94
glyma.06g118500
glyma.06g118600
50.72
44.68
glyma.08g109200
glyma.08g109300
glyma.08g109400
glyma.08g109500
glyma.08g110300
glyma.08g110400
glyma.08g110500
glyma.08g110700
glyma.08g110900
47.00
glyma.09g074900
glyma.09g075200
46.84
50.97
Glma-chr11.map
Glma-chr12.map
Glma-chr13.map
Glma-chr14.map
Glma-chr15.map
Glma-chr16.map
Glma-chr17.map
Glma-chr18.map
Glma-chr19.map
Glma-chr20.map
glyma.11g011500
glyma.11g097900
39.17
glyma.12g023800
40.11
glyma.13g034300
44.41
49.71
50.94
37.40
41.91
62.31
glyma.19g105100
50.59
glyma.20g224500
46.77
25
50
cM

Medicago truncatula

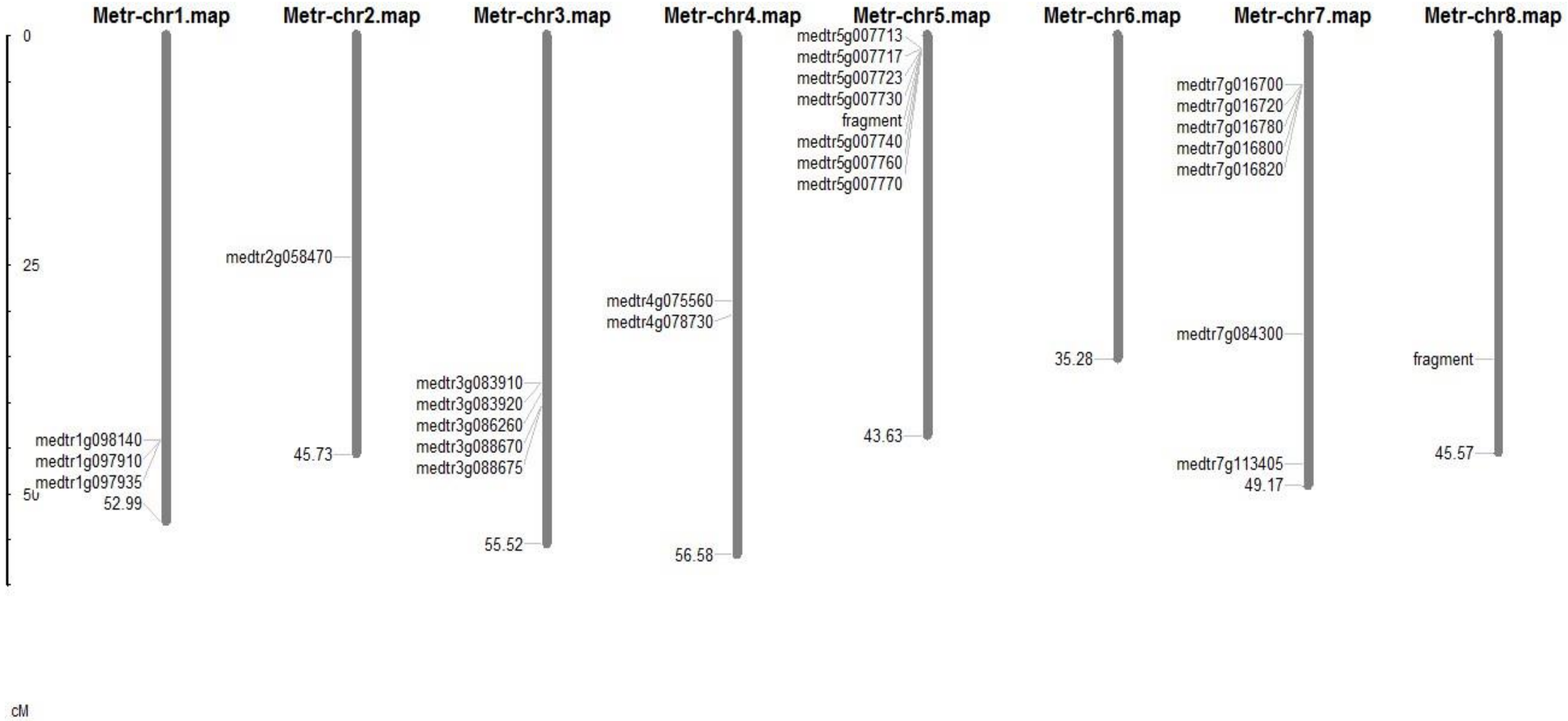

Physcomitrella patens

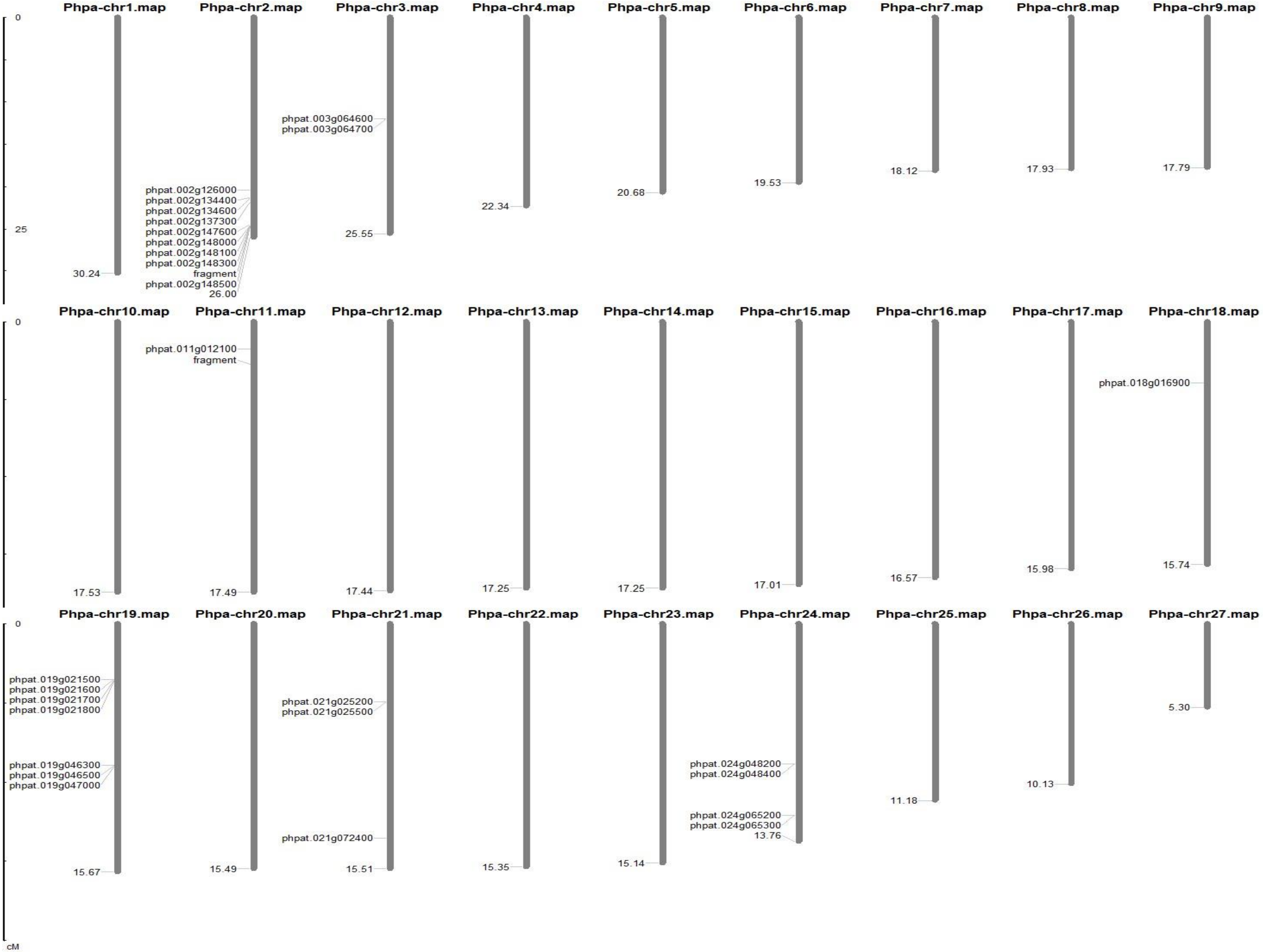

Populus trichocarpa

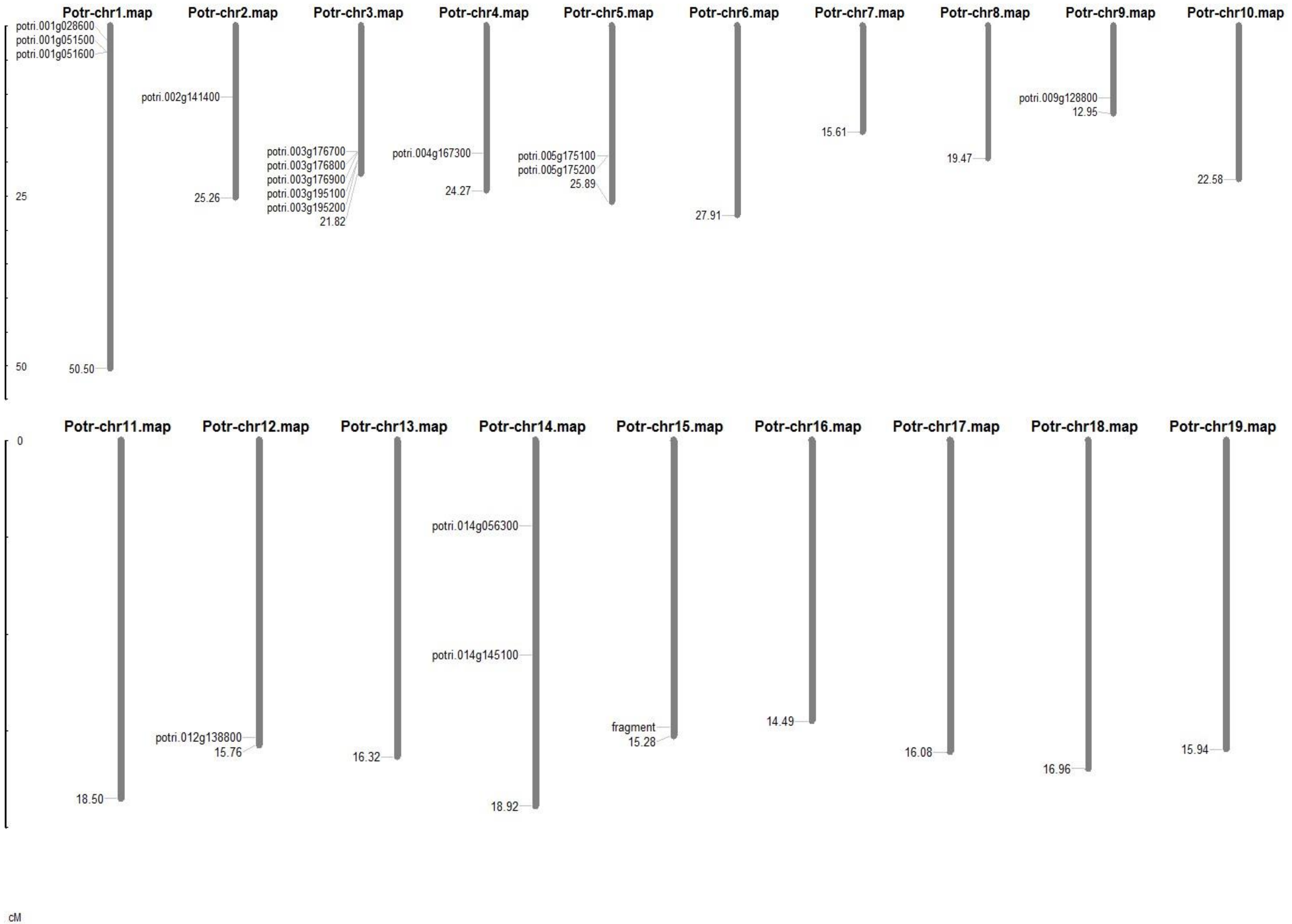

Solanum lycopersicum

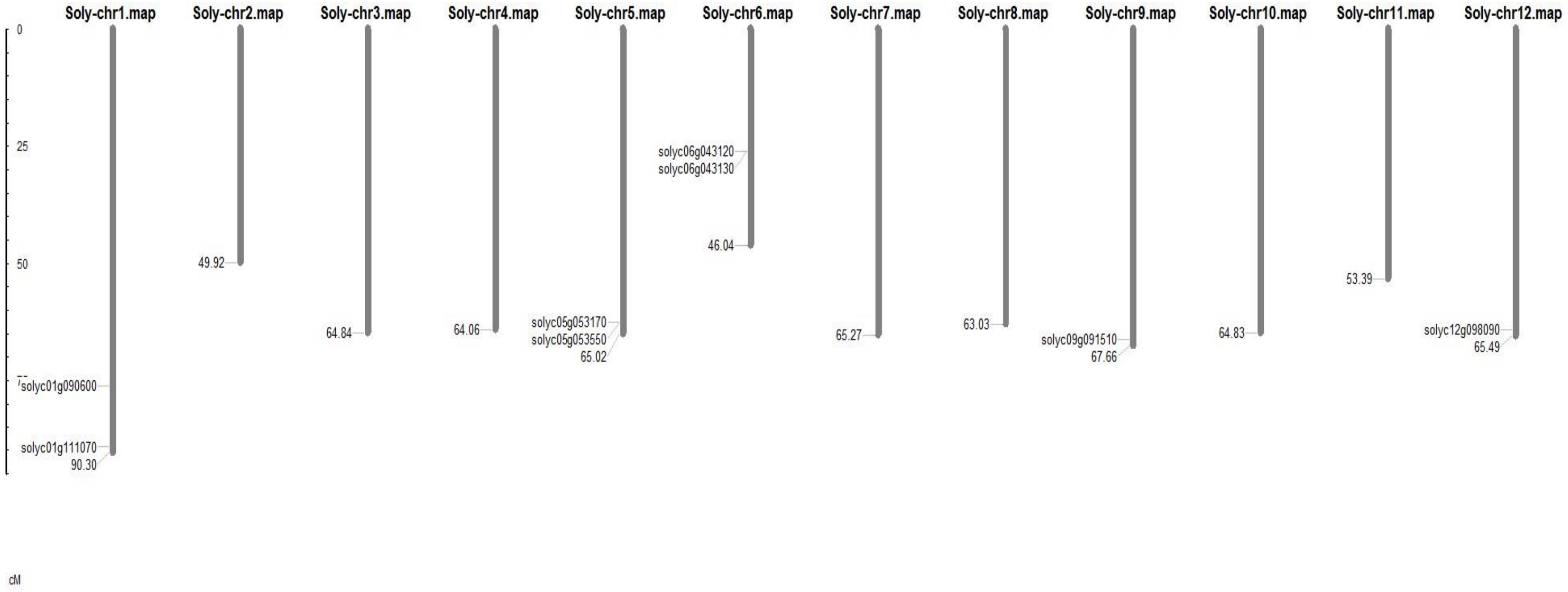

Solanum tuberosum

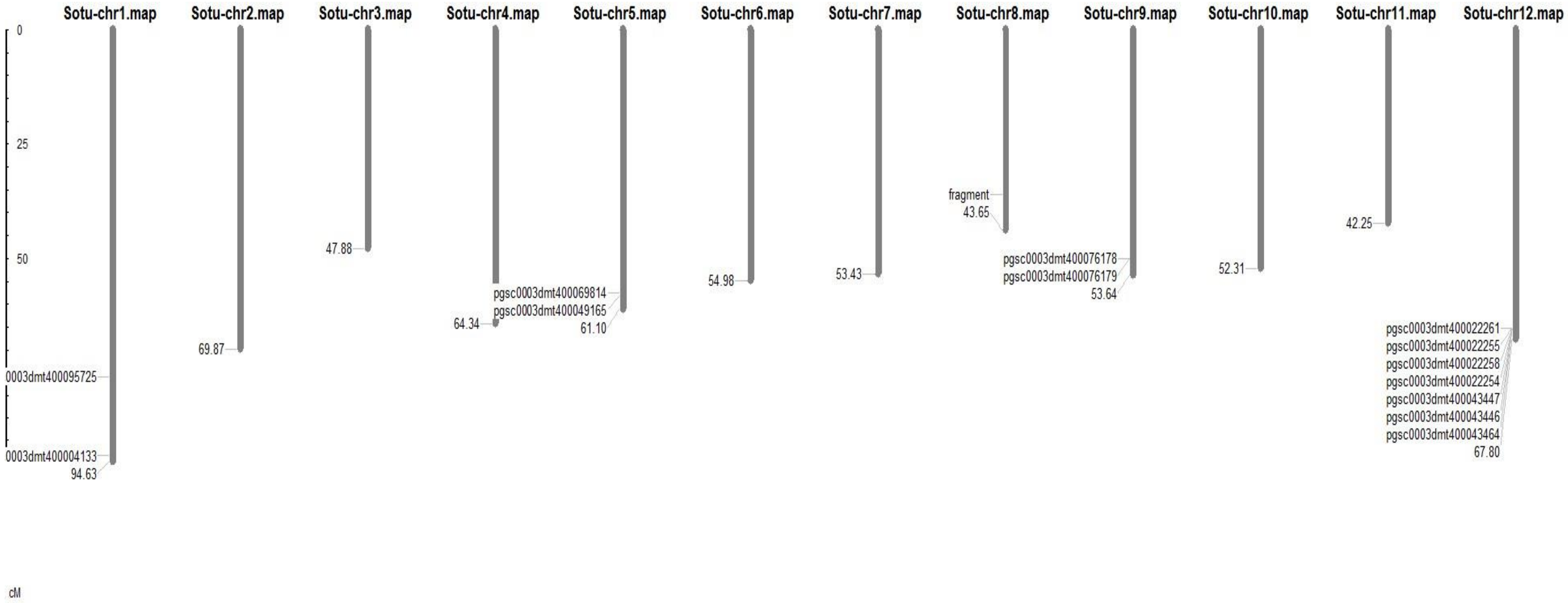

Vitis vinifera

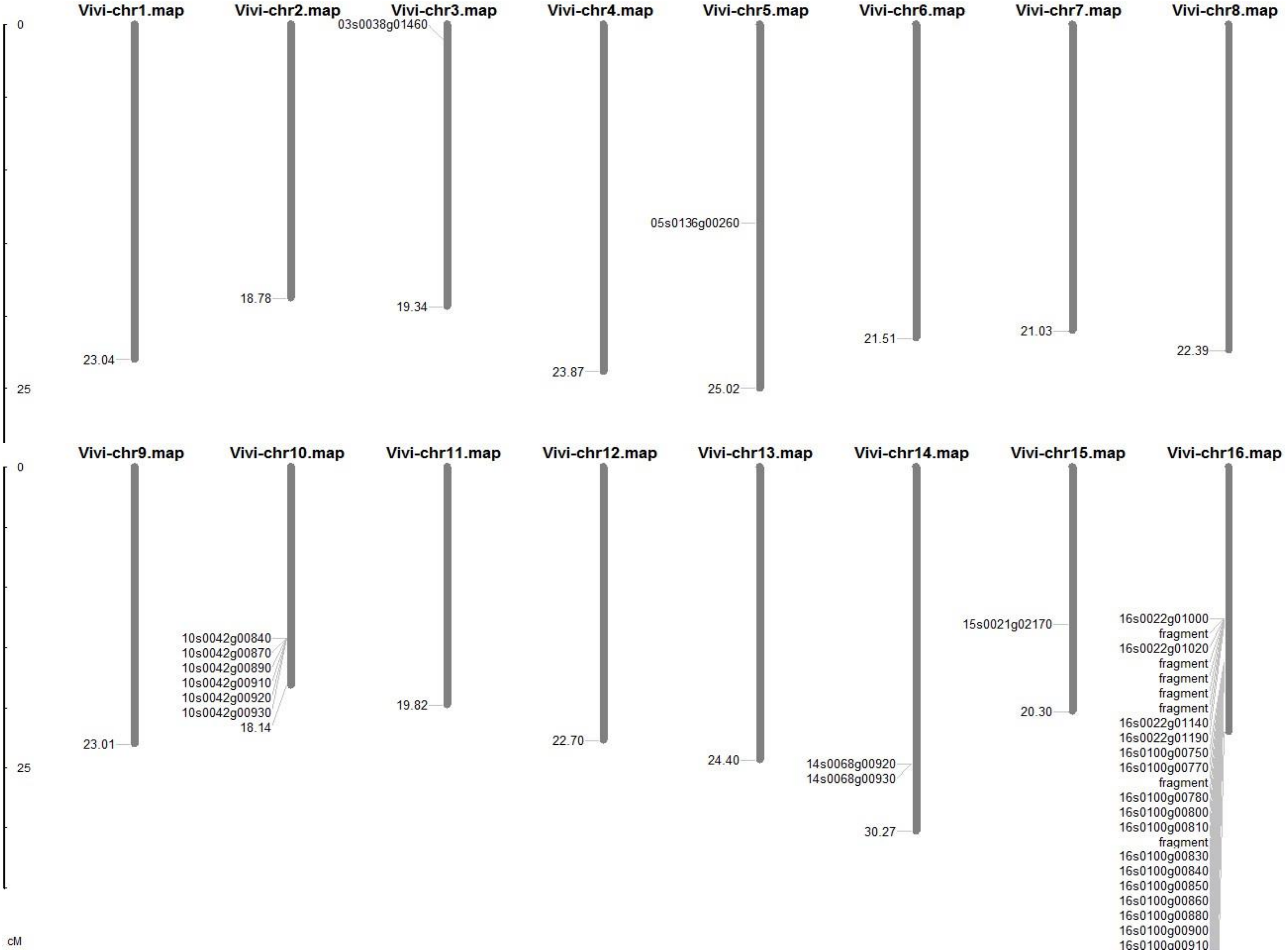

Citrus clementina

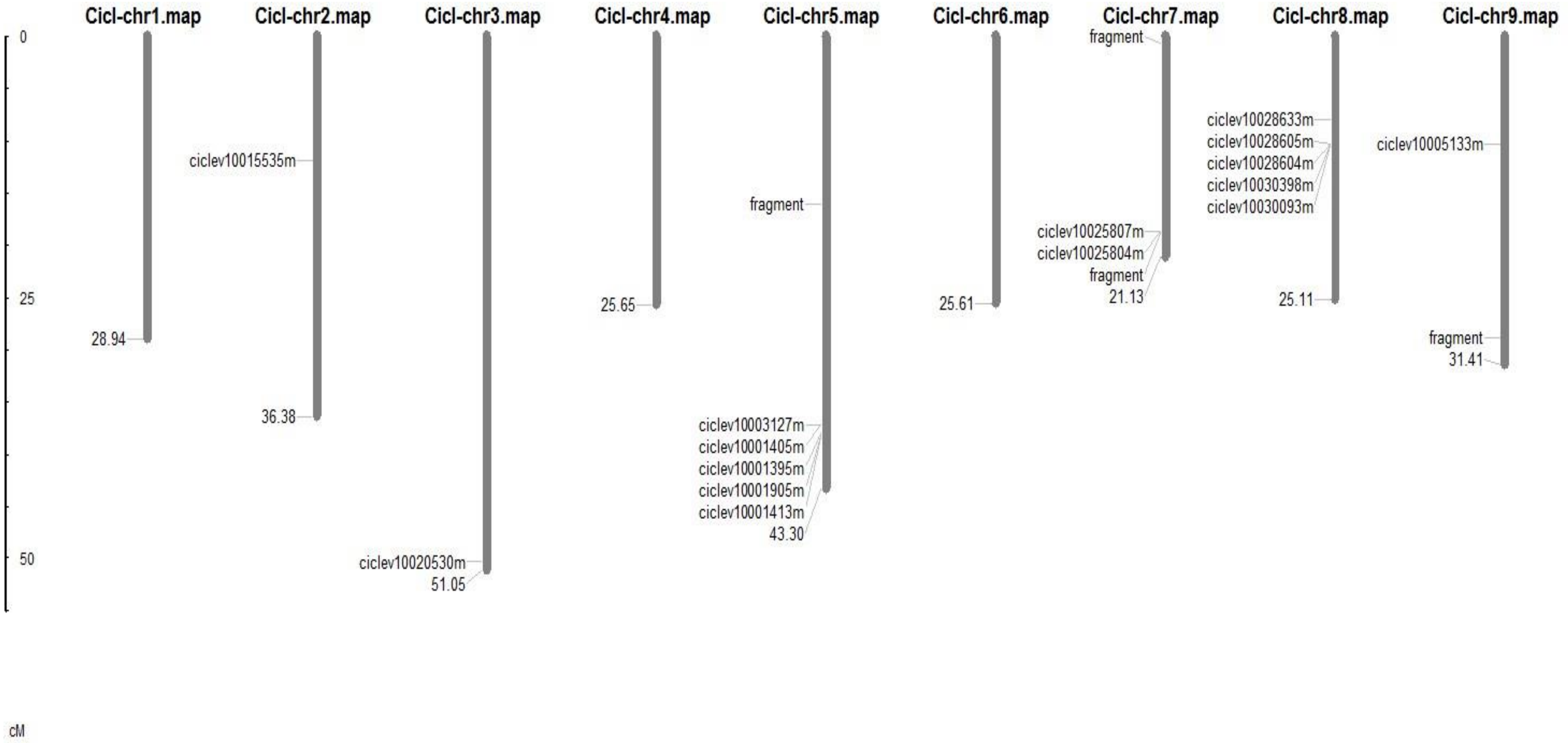

Oryza sativa

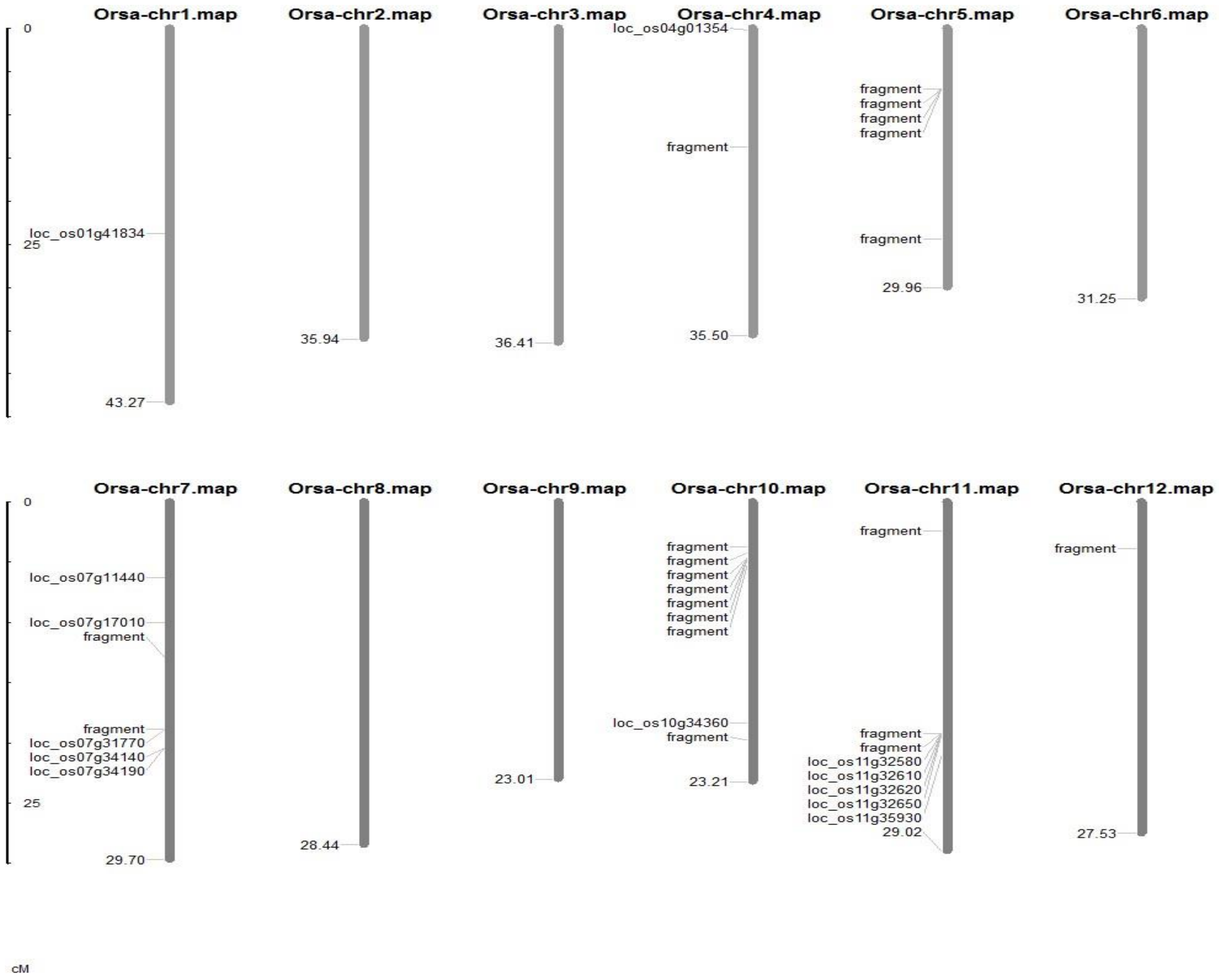

Zea mays

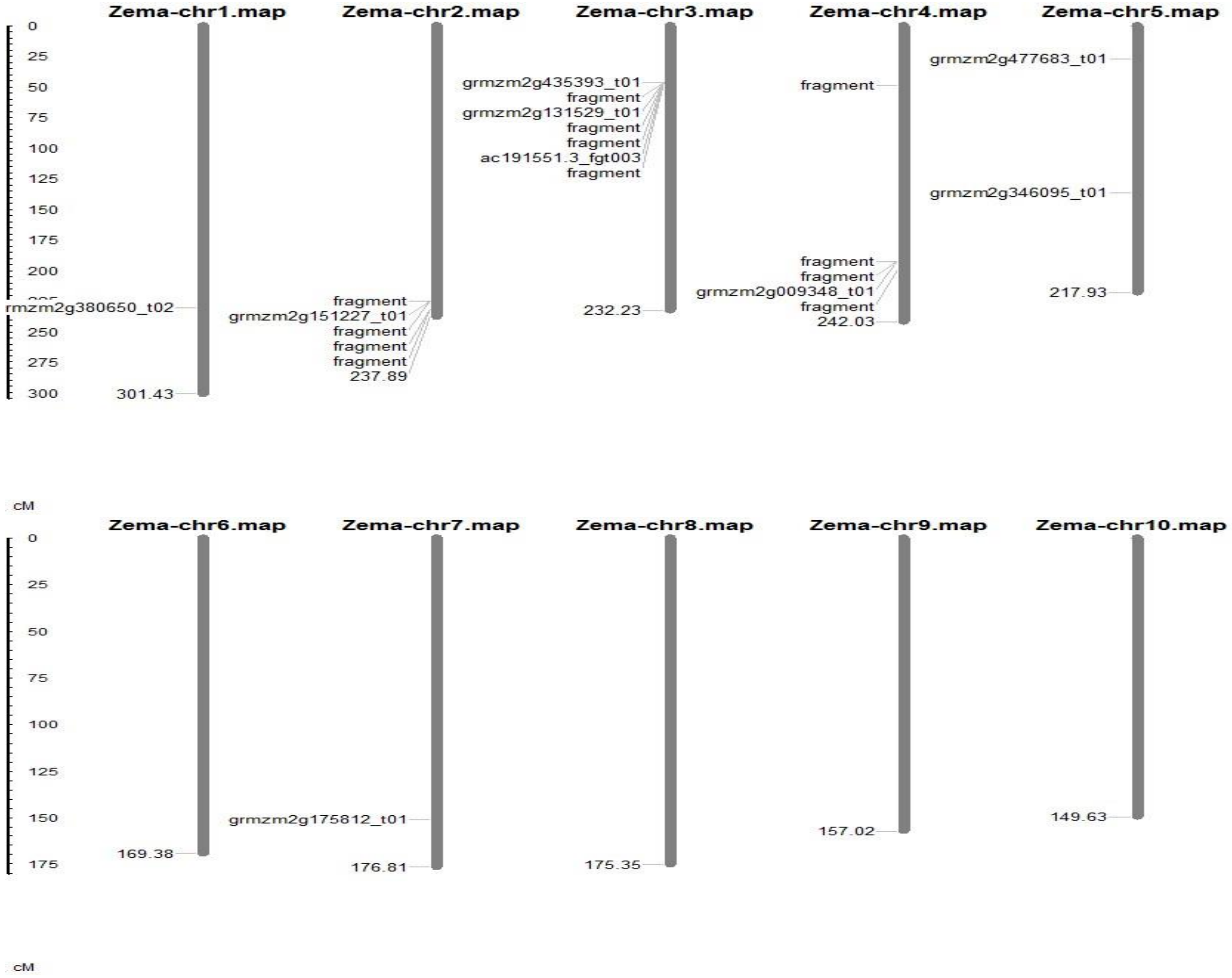

Zema-chr1.map
Zema-chr2.map
Zema-chr3.map
Zema-chr4.map
Zema-chr5.map
0
25
50
75
100
125
150
175
200
250
275
300
rmzm2g380650_t02
301.43
fragment
grmzm2g151227_t01
fragment
fragment
fragment
237.89
grmzm2g435393_t01
fragment
grmzm2g131529_t01
fragment
fragment
ac191551.3_fgt003
fragment
232.23
fragment
fragment
fragment
grmzm2g009348_t01
fragment
242.03
grmzm2g477683_t01
grmzm2g346095_t01
217.93
cM
Zema-chr6.map
Zema-chr7.map
Zema-chr8.map
Zema-chr9.map
Zema-chr10.map
0
25
50
75
100
125
150
175
169.38
grmzm2g175812_t01
176.81
175.35
157.02
149.63
cM

Supplemental Figure 2

Ancestral state reconstruction of eight positive sites (121, 208, 264, 265, 266, 276, 300, 340) in branch a6'.

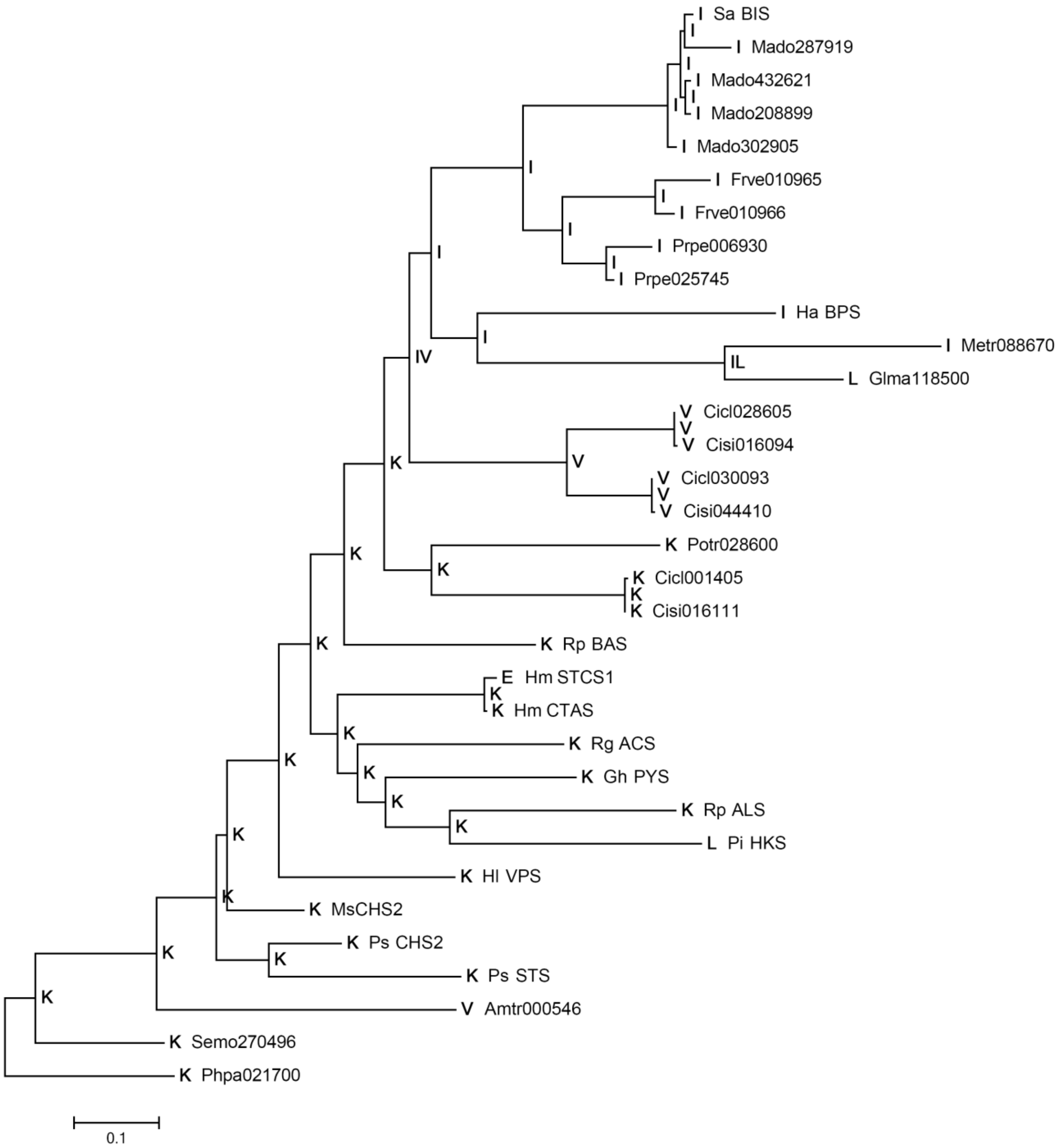

121 K→I

208 S→V

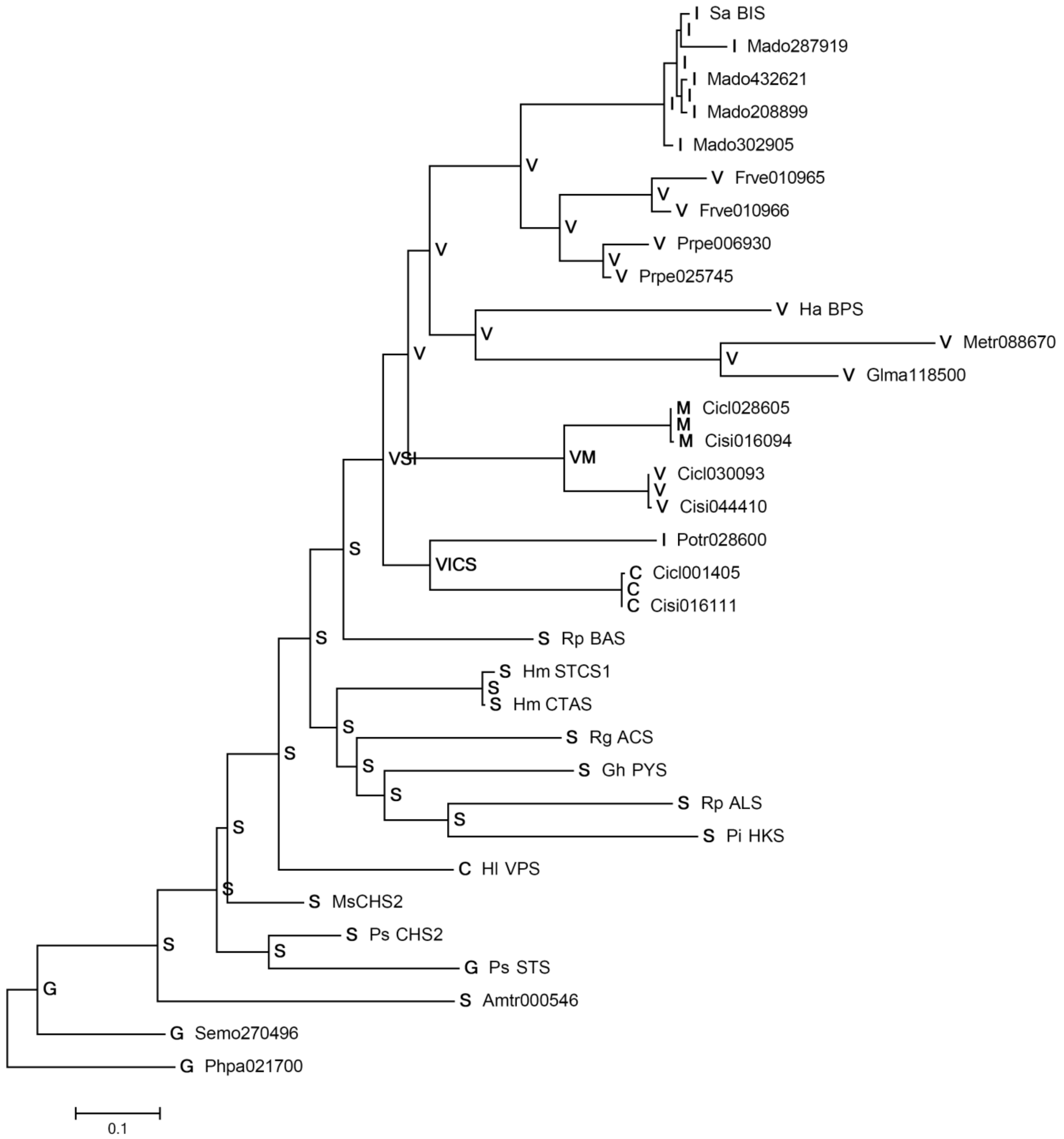
Sa BIS
Mado287919
Mado432621
Mado208899
Mado302905
Frve010965
Frve010966
Prpe006930
Prpe025745
Ha BPS
Metr088670
Glma118500
Cicl028605
Cisi016094
Cicl030093
Cisi044410
Potr028600
Cicl001405
Cisi016111
Rp BAS
Hm STCS1
Hm CTAS
Rg ACS
Gh PYS
Rp ALS
Pi HKS
Hl VPS
MsCHS2
Ps CHS2
Ps STS
Amtr000546
Semo270496
Phpa021700
VSI
VM
VICS
0.1

264 T→E

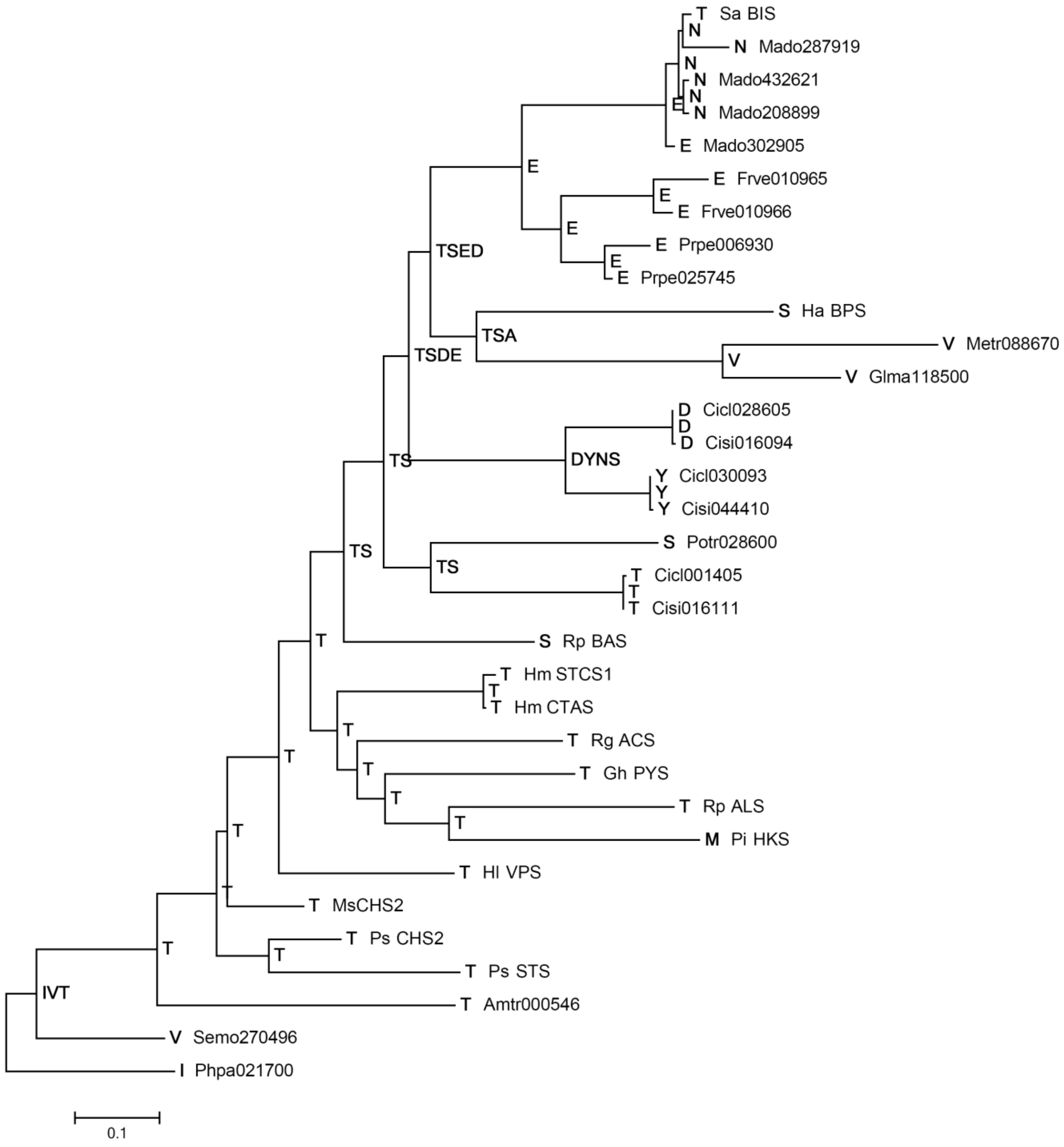
T Sa BIS
N
N Mado287919
N
N Mado432621
N
E
N Mado208899
E Mado302905
E
E Frve010965
E
E Frve010966
E
E Prpe006930
E
E Prpe025745
TSED
S Ha BPS
TSA
V Metr088670
V
V Glma118500
TSDE
D Cicl028605
D
D Cisi016094
DYNS
TS
Y Cicl030093
Y
Y Cisi044410
S Potr028600
TS
T Cicl001405
T
T Cisi016111
TS
S Rp BAS
T
T Hm STCS1
T
T Hm CTAS
T
T Rg ACS
T
T Gh PYS
T
T Rp ALS
T
M Pi HKS
T
T Hl VPS
T
T MsCHS2
T Ps CHS2
T
T Ps STS
T
T Amtr000546
IVT
V Semo270496
I Phpa021700
0.1

265 F→Y

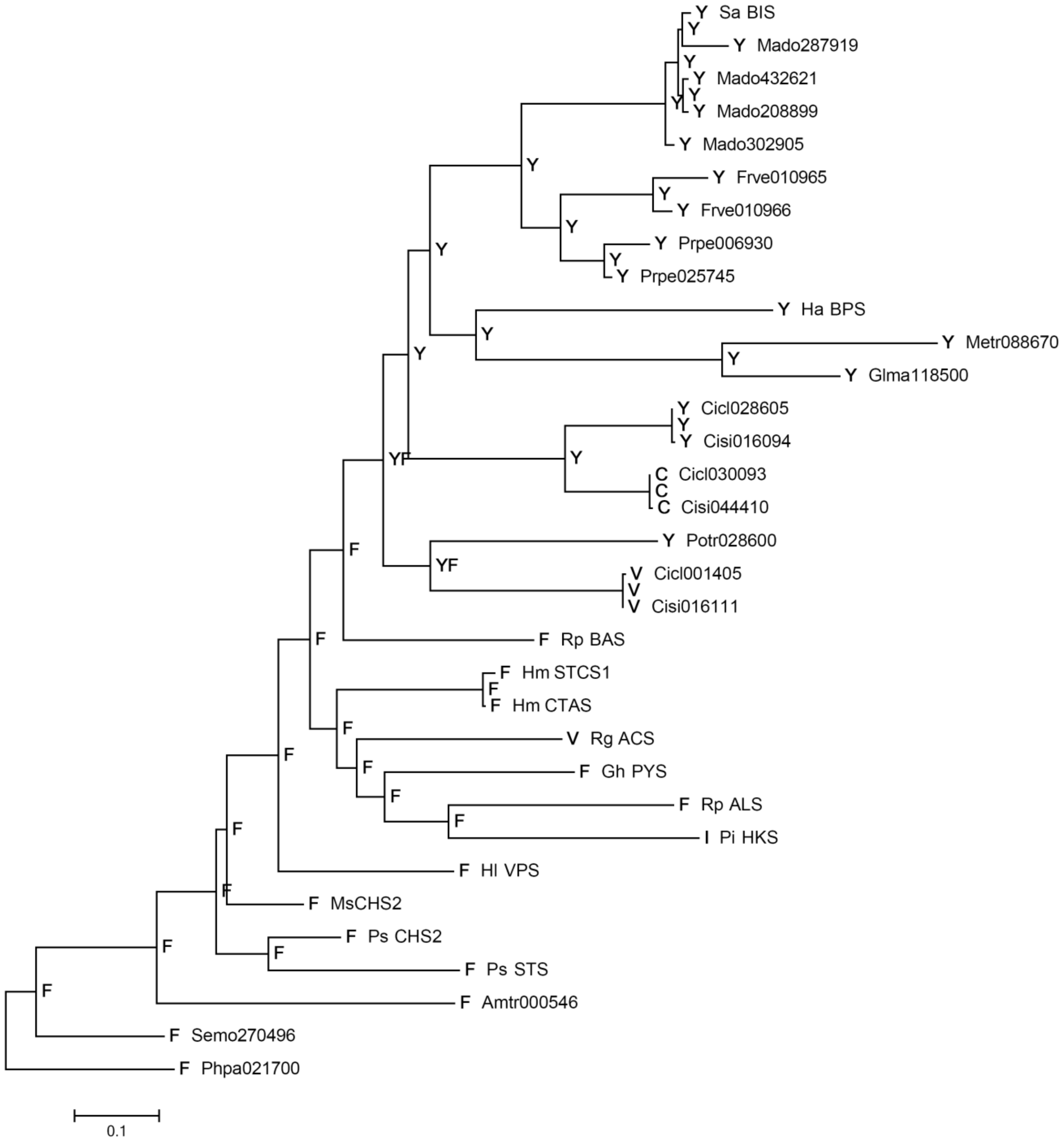

Y Sa BIS
Y Mado287919
Y Mado432621
Y Mado208899
Y Mado302905
Y Frve010965
Y Frve010966
Y Prpe006930
Y Prpe025745
Y Ha BPS
Y Metr088670
Y Glma118500
Y Cicl028605
Y Cisi016094
C Cicl030093
C Cisi044410
Y Potr028600
V Cicl001405
V Cisi016111
F Rp BAS
F Hm STCS1
F Hm CTAS
V Rg ACS
F Gh PYS
F Rp ALS
I Pi HKS
F Hl VPS
F MsCHS2
F Ps CHS2
F Ps STS
F Amtr000546
F Semo270496
F Phpa021700
0.1

266 H→Y

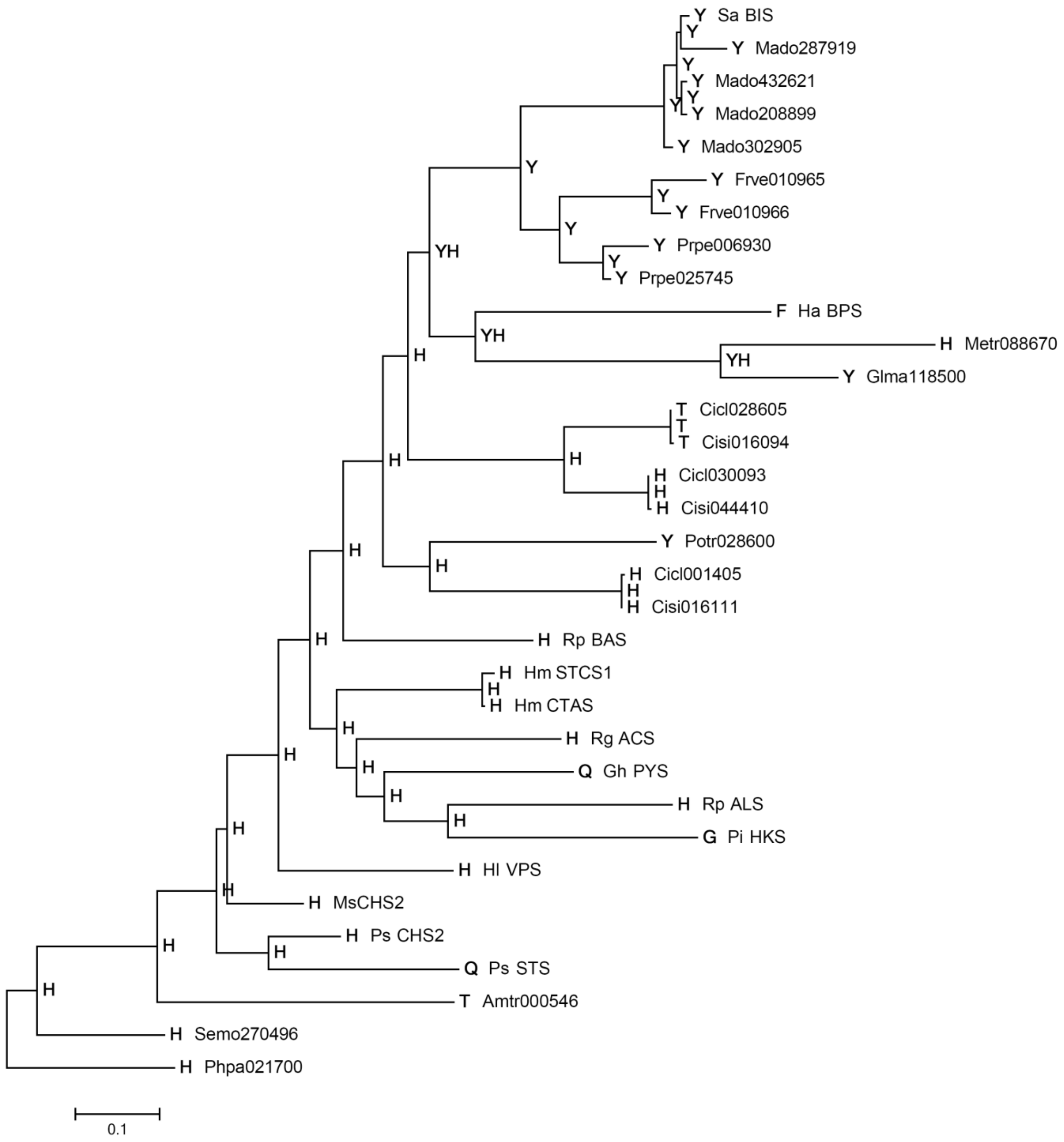
Y Sa BIS
Y Mado287919
Y Mado432621
Y Mado208899
Y Mado302905
Y Frve010965
Y Frve010966
Y Prpe006930
Y Prpe025745
F Ha BPS
H Metr088670
Y Glma118500
T Cicl028605
T Cisi016094
H Cicl030093
H Cisi044410
Y Potr028600
H Cicl001405
H Cisi016111
H Rp BAS
H Hm STCS1
H Hm CTAS
H Rg ACS
Q Gh PYS
H Rp ALS
G Pi HKS
H Hl VPS
H MsCHS2
H Ps CHS2
Q Ps STS
T Amtr000546
H Semo270496
H Phpa021700
YH
YH
YH
0.1

276 S→G

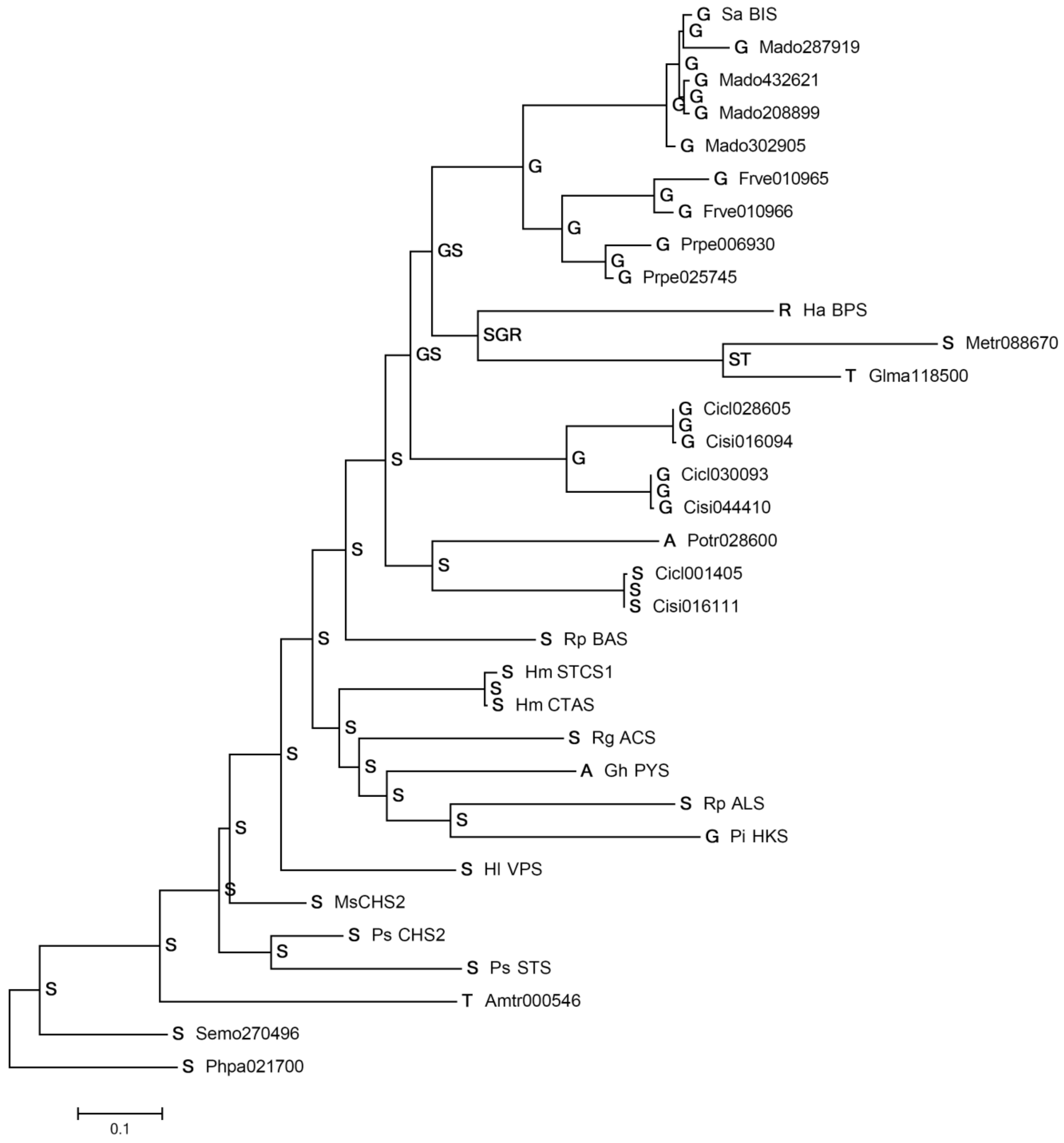

G Sa BIS
G Mado287919
G Mado432621
G Mado208899
G Mado302905
G Frve010965
G Frve010966
G Prpe006930
G Prpe025745
R Ha BPS
SGR
S Metr088670
ST
T Glma118500
GS
G Cicl028605
G Cisi016094
G Cicl030093
G Cisi044410
A Potr028600
S Cicl001405
S Cisi016111
S Rp BAS
S Hm STCS1
S Hm CTAS
S Rg ACS
A Gh PYS
S Rp ALS
G Pi HKS
S Hl VPS
S MsCHS2
S Ps CHS2
S Ps STS
T Amtr000546
S Semo270496
S Phpa021700
0.1

300 W→Y

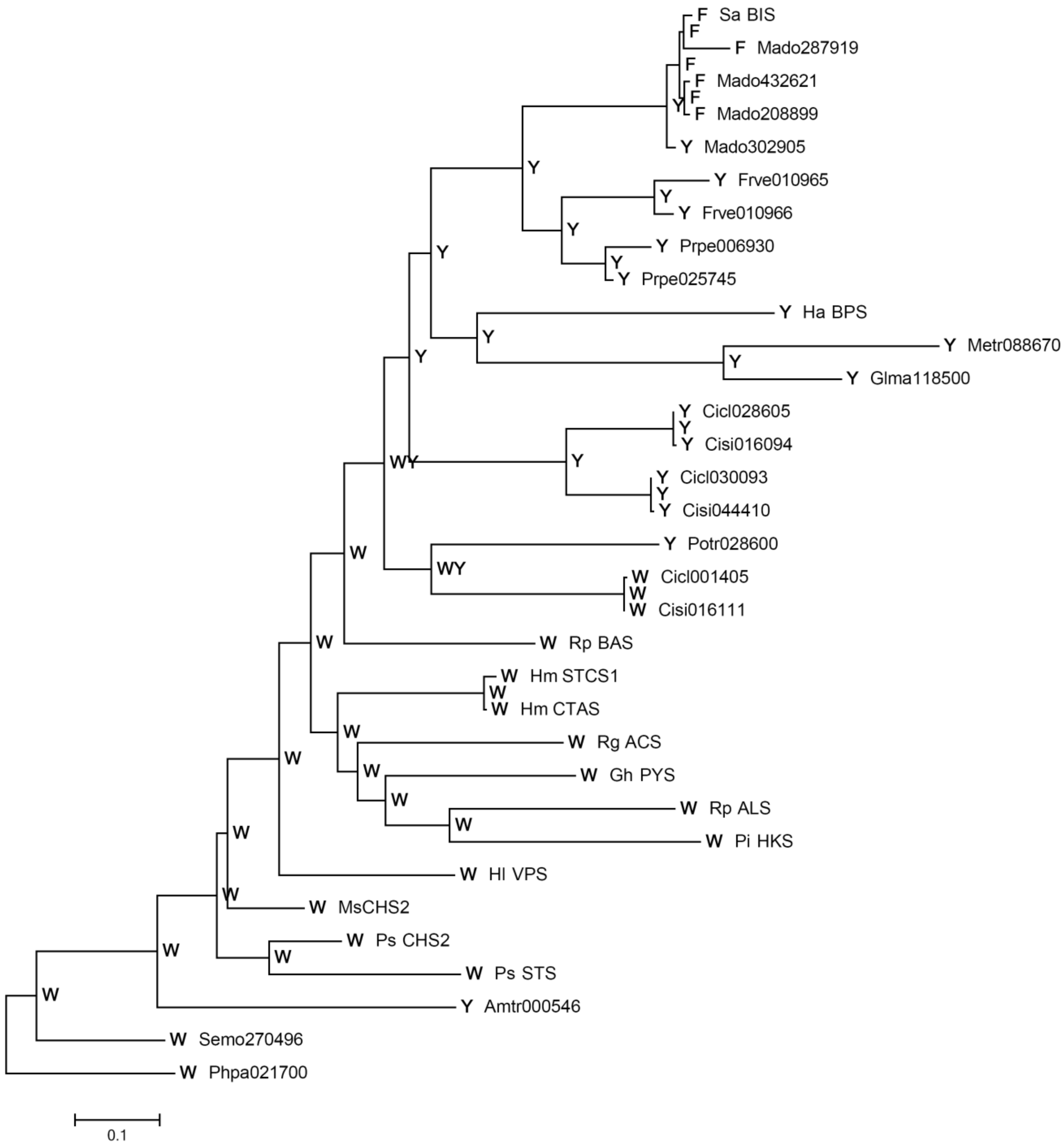

F Sa BIS
F Mado287919
F Mado432621
F Mado208899
Y Mado302905
Y Frve010965
Y Frve010966
Y Prpe006930
Y Prpe025745
Y Ha BPS
Y Metr088670
Y Glma118500
Y Cicl028605
Y Cisi016094
Y Cicl030093
Y Cisi044410
Y Potr028600
W Cicl001405
W Cisi016111
W Rp BAS
W Hm STCS1
W Hm CTAS
W Rg ACS
W Gh PYS
W Rp ALS
W Pi HKS
W Hl VPS
W MsCHS2
W Ps CHS2
W Ps STS
Y Amtr000546
W Semo270496
W Phpa021700
0.1

340 A→P

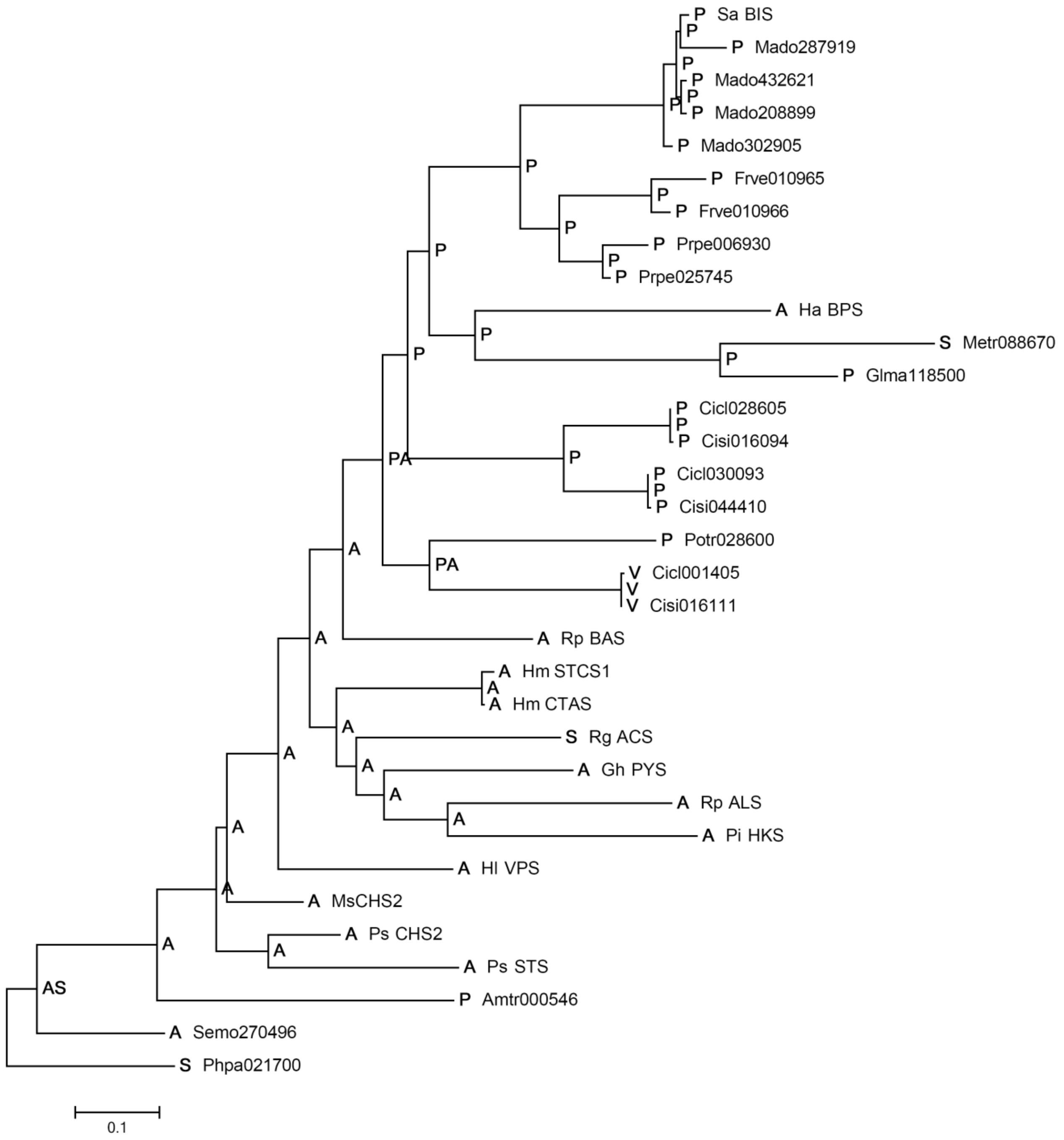

P Sa BIS
P Mado287919
P Mado432621
P Mado208899
P Mado302905
P Frve010965
P Frve010966
P Prpe006930
P Prpe025745
A Ha BPS
S Metr088670
P Glma118500
P Cicl028605
P Cisi016094
P Cicl030093
P Cisi044410
P Potr028600
V Cicl001405
V Cisi016111
A Rp BAS
A Hm STCS1
A Hm CTAS
S Rg ACS
A Gh PYS
A Rp ALS
A Pi HKS
A Hl VPS
A MsCHS2
A Ps CHS2
A Ps STS
P Amtr000546
A Semo270496
S Phpa021700
PA
AS
0.1
